# We’re All the Same: Collective Narcissists’ Cross-National Support for Putin and Russian Military Attacks

**DOI:** 10.5334/irsp.761

**Published:** 2024-01-17

**Authors:** Genavee Brown, Gaëlle Marinthe

**Affiliations:** 1 Northumbria University, UK; 2 Université Paris 8 Vincennes-Saint-Denis, FR

**Keywords:** collective narcissism, similarity, Russia, Ukraine, political psychology

## Abstract

National narcissism is associated with support for nationalist and anti-democratic leaders and decisions in one’s own country. We hypothesize that it might also relate to more favorable judgments of outgroup nationalist leaders and actions, even if the latter may pose a threat to the ingroup. Using the context of the Russian attack on Ukraine, we hypothesize that people with a higher level of national narcissism would be more supportive of Russian attacks, Russian President Vladimir Putin, and the Russian people. This may be due to a higher perception of belief similarity to Putin and Russians. We also considered the moderating role of the explicit target of the attack (Ukraine vs. ingroup). We tested our hypotheses in two studies (Study 1: *N* = 339 French; Study 2: *N* = 400 Americans). In both studies, national narcissism was related to a judgment of the attack (on Ukraine or the ingroup) as less immoral and to a better opinion of Putin. These effects were mediated by perceived belief similarity. In both studies however, these less negative judgments of the attack and of Putin did not extend to Russian people. Our results highlight that national narcissists are inclined to support a nationalist outgroup leader and their violent actions, although these may ultimately harm the ingroup.

On 24 February 2022, Russian President Vladimir Putin announced an attack on Ukraine by Russian armed forces. Political leaders in Europe and the United States (US) largely condemned Vladimir Putin and called for support for Ukraine (The Economist, 2022). However, before the attack on Ukraine, some citizens and political leaders outside of Russia expressed support for Putin, despite his previous military attacks suggesting his tendencies toward aggressive leadership (e.g., in Crimea in 2014). In France, for example, Jean-Luc Mélenchon (leader of the far-left party) and Marine le Pen (leader of the far-right party) have regularly spoken out in favor of a rapprochement with Putin’s Russia (Franceinfo, 2022). Additionally, some politicians even refused to support Ukraine or condemn the Russian attack, such as the 57 US Republican senators who refused to vote on a bill to financially support Ukraine (Desiderio, 2022). Thus, while among political leaders and citizens in Europe and North America, support for Ukraine has been predominant, some discourse defending Putin’s Russia against Ukraine has emerged.

This research examines the identity factors that may be associated with a more positive judgment of Putin and Russian military attacks. We argue that a defensive form of national identity (national collective narcissism) could be associated with more favorable attitudes towards Putin and a perception of Russian attacks as more moral, particularly when an outgroup (in this case Ukraine) is targeted.

## National Narcissism, Nationalism, and Anti-Democratic Actions

In the psychosocial tradition, mere membership or identification with a social group has been identified as a major predictor of intra- and intergroup attitudes and behaviors (Tajfel & Turner, 1979). However, beyond the strength of identification, subsequent research has highlighted the importance of different forms of group identity in understanding intra- and intergroup processes (e.g., Golec de Zavala et al., 2009; Kosterman & Feshbach, 1989; Roccas et al., 2006; Schatz et al., 1999). We draw here on the theoretical field of collective narcissism, a defensive form of identity defined as the belief in the greatness of one’s group being insufficiently recognized by others (Cichocka, 2016; Cichocka & Cislak, 2020; Golec de Zavala et al., 2009). It differs from secure identity, referring to an investment in the ingroup devoid of this quest for recognition and grandeur (Cichocka, 2016; Cichocka & Cislak, 2020). At the national level, collective narcissism (i.e., national narcissism) therefore corresponds to a need for recognition of one’s nation, which in certain contexts takes the form of a search for prestige and/or a need for dominance, also known as nationalism^1^ (Cichocka & Cislak, 2020; Kosterman & Feshbach, 1989).

National narcissism is defensive in that it acts in a compensatory way, as a reaction to collective deprivation (Marchlewska et al., 2018) or to frustrated individual needs (e.g., lack of personal control, Bertin et al., 2022; Cichocka et al., 2018; low self-esteem, Golec de Zavala et al., 2020). This belief has consequences at the political level (Cichocka & Cislak, 2020), such as stronger support for nationalism and anti-democratic leaders and policies (Marchlewska et al., 2018). For example, people with higher levels of national narcissism in the US were more likely to support Donald Trump (Federico & Golec de Zavala, 2018), the nationalist ultraconservative Law and Order party in Poland (Marchlewska et al., 2018), Viktor Orbán’s Fidesz populist party in Hungary (Lantos & Forgas, 2021), or to vote for Brexit in Britain which promoted nationalist ideals of independence and dominance (Marchlewska et al., 2018). In this respect, nationalism is close to, and shares rhetoric with, other concepts such as national narcissism (Cichocka & Cislak, 2020), that defend a single and narrow definition of a (grandiose) nation alongside a desire for national domination. Both national narcissism and nationalism, in their willingness to gain status and/or power for the ingroup, may lead to support for undemocratic actions and policies, such as the attack on the Capitol in the US (Keenan & Golec de Zavala, 2021) or secret surveillance of their country’s citizens (Biddlestone et al., 2022).

In sum, people with high levels of national narcissism are more likely to support nationalist leaders and policies in their country, as they may fulfill the recognition-seeking nature of national narcissists. However, we are interested here in a different situation: support for nationalist actions, namely invading other nation states, by *outgroup* leaders, who are therefore not a priori defending the interests of the ingroup and may even be threatening.

## National Narcissism and Support for Anti-Democratic Outgroup Leaders and Actions

Although a plethora of research indicates that national narcissists are inclined to support hostile attitudes and policies towards outgroups seen as threatening (e.g., Golec de Zavala et al., 2013, 2016; Guerra et al., 2020; Jasko et al., 2017; Cichocka et al., 2022; Kazarovytska & Imhoff, 2022), this may not always be the case. While some outgroups may constitute a threat to the ingroup, research suggests that collective narcissists may support an outgroup if it benefits the ingroup. For example, American national narcissists judged remaining an ally with Saudi Arabia after the murder of journalist Jamal Khashoggi to be more moral when it was presented as benefiting the US than when it was not (Bocian et al., 2021). However, the question remains unanswered whether such support for violent, outgroup actions could be expressed if ingroup interests are not at stake. We suggest that national narcissism may be related to support for an outgroup nationalist leader and violent actions, even when the context does not benefit the ingroup, due to a perception of belief similarity.

Belief similarity is a major factor in interpersonal judgment (Montoya & Horton, 2013) and political attitudes such as voting (e.g., Caprara et al., 2007). Observers judge individuals perceived to have the same beliefs more positively (both on morality and competence; Bocian et al., 2018). Similarly, people display less prejudice towards groups that are perceived as having similar values (e.g., Wolf et al., 2021). This can be observed in the situation with Russia’s and Putin’s Western supporters. Braghiroli and Makarychev (2016) define Putin’s rhetoric as trans-ideological in the sense that it goes beyond the left/right political dichotomy. These authors state that this rhetoric is based on four fundamental points: conservative values, a defense of sovereignty and national interest, an anti-US liberalism stance, and a glorification of the country’s Soviet past. Ultimately, these core components of Putin’s rhetoric may be an attempt to fulfill a nationalist goal of regaining dominance in global politics (Wright, 2022). Ideological similarity on some of these core aspects partly explains Western far-left and far-right political leaders’ support for Putin (Braghiroli & Makarychev, 2016). Similarly, Putin’s rhetoric may appeal to those high in national narcissism. Nationalist rhetoric, defending a glorified nation against enemies, resonates with national narcissistic beliefs (e.g., Golec de Zavala & Bierwiaczonek, 2021). Furthermore, the will to gain power (e.g., Cichocka & Cislak, 2020) and conservative values (e.g., Lantos & Forgas, 2021; Marinthe et al., 2022) are also strongly associated with national narcissists’ beliefs. We thus argue that national narcissists (even outside of Russia) may perceive themselves as similar to Putin in terms of identity-related beliefs. As for interpersonal relations, this perception of similarity may lead national narcissists to express better attitudes towards Putin and Russia’s actions, here, an attack on another country.

While national narcissists may be open to supporting an attack on another country made by those with similar beliefs to themselves, they are hypersensitive to criticism and threat to their own country and are likely to express hostility towards groups seen as threatening their national identity (e.g., Golec de Zavala et al., 2013, 2016). Notably, Putin has pointed directly to the European Union (EU) and the US as enemies and potential targets of attacks in his rhetoric (Braghiroli & Makarychev, 2016) and during the Ukraine conflict (Gibert, 2022).

While national narcissists may focus on perceived belief similarity when the threat explicitly targets an outgroup, this effect could be attenuated when the attack explicitly targets the (national) ingroup. Namely, an attack on the ingroup would constitute a threat to the image of the group and, regardless of similarity with the attacking nation state or their leader, national narcissists would most likely judge the attack as less moral and perceive the leader less positively.

## Overview

In this research we focus on a form of national identity (national narcissism) that may explain support for an outgroup nationalist leader and his actions by relying on the context of the military attacks in Ukraine by Putin’s Russia. We examine moral judgments of the conflict, judgments of Putin, and the Russian people in two countries: France and the US.

We chose France and the US as target countries based on the differences in their proximity both physically and diplomatically to Russia, which we outline below. France’s recent history with Russia relations were strengthened during World War I (WWI) and World War II (WWII) when they fought together against Germany in the first half of the 20th century. France and Russia had diplomatic ties after WWII, and diplomacy between the countries continues today, although Franco-Russian tensions have returned as Russia began the invasion of Donbas (2014) and Ukraine (2022). Indeed, French news outlets reported on the potential for the conflict in Ukraine to expand to the rest of continental Europe in the early days of the war (Le Monde, 2022).

The US relationship with Russia has been more conflictual. During WWI, the US was allied with Russia but only hesitantly so due to their human rights abuses against non-ethnic Russians (e.g., Jews, Finns, Eastern Europeans). After WWI, when the Soviet Union formed, the relations between the US and Soviet Union deteriorated into the ideological conflict between liberal democracy and communism that spawned the Cold War after WWII. Although the Cold War has ended, diplomatic visits between the countries are still rare and tensions continue. When the conflict in Ukraine began, there was little talk of war on US soil but support for Ukraine stemmed from the long-standing tensions between the two countries.

Thus, while France has had a relatively less conflictual political history with Russia, their physical proximity to the current conflict in Ukraine means that the conflict poses an existential threat to the country. In the US, there is little talk of a physical attack, but the conflict has resurrected political and ideological tensions that date from the Cold War. Thus, these countries provide the chance to test views on the conflict from two different political contexts and consistency across them.

Across two studies, we hypothesize that individuals with higher levels of national narcissism would be more likely to judge Russian attacks on other countries as moral and to judge Putin more positively. We hypothesize that this positive link will be explained by perceived belief similarity about one’s type of national identification and that it will be accentuated when the target of the attack is an outgroup (Ukraine) rather than the ingroup. We also explored whether the more positive evaluation of the attack and of Putin could extend to the Russian people, by looking at the link between national narcissism and judgments of the Russian people.

Studies 1 (conducted in France) and 2 (conducted in the US) measured judgment of the attack, Putin, and the Russian people, as well as the perception of identity-related belief similarity. Studies 1 and 2 also manipulated the target of the attack (Ukraine vs. ingroup: France or US, respectively). In both studies, analyses that test for robustness of results, by controlling for national satisfaction, can be found in Supplemental Material.

Data and scripts for Studies 1 and 2 are available on the OSF (https://osf.io/unkxy/). Studies 1 and 2 were reviewed and given ethical approval through the Northumbria University Ethics Approval System, reference number 44940.

## Study 1

In Study 1, we hypothesize a link between national narcissism and support for Russia and Putin (on measures of moral judgment of a Russian military attack on Ukraine or France and social judgment of Putin). We also examined if these more positive attitudes towards Putin could extend to the Russian people. Moreover, we further examined the potential moderating role of the target of the attack: support for Russia and Putin from those with higher levels of national narcissism should be greater when Russian attacks target an outgroup (Ukraine) than an ingroup (France). These hypotheses were pre-registered as main hypotheses (https://osf.io/m3nzy). Finally, we examine the mediating effect of perceived similarity with Putin and Russians in the link between national narcissism and support for Russia and Putin (pre-registered as exploratory models).

### Method

#### Participants

We recruited 360 participants on various social media sites (Facebook, Twitter, Reddit) in groups related to politics (from all sides of the political spectrum, e.g., ‘Macron nous t’aimons’, ‘Mélenchon 2022’, and ‘je suis FN/RN’), news (e.g., ‘Politique 2022’), student life (e.g., ‘étudiants de Rennes’), and mutual aid (e.g., ‘Wanted Community Paris’), from 10 March to 19 May 2022. As pre-registered, we excluded participants who were not French (*n* = 18), who did not answer both attention checks correctly (*n* = 5), and who failed to imagine a Russian attack on France (*n* = 1). The final sample consisted of 339 participants (144 men, 187 women, 3 non-binary, 5 missing), aged 18 to 80 (*M* = 37.11, *SD* = 17.17). Participants were slightly left-wing (*M* = 3.26, *SD* = 1.69, on a scale ranging from 1 to 7 with higher scores indicating a more right-wing political orientation).^2^

#### Procedure

Participants were invited to take part in a study examining whether beliefs about one’s country influence foreign policy beliefs. First, participants completed measures on national narcissism, national satisfaction, and similarity to Putin and the Russian people.

Next, participants read a short article reminding them of the facts related to the Russian military attack in Ukraine (condition *Ukraine target*; the complete induction can be found in Supplemental Material) which was inspired by a French Associated Press report. We chose to have all participants view the Ukraine target condition first because we needed to remind participants of the details of the attack on Ukraine before asking them to imagine a similar attack on their home soil. After reading the article about the attack in Ukraine, participants completed measures of judgment of the attack in Ukraine (as moral), judgment of Putin, and a feeling thermometer on different national groups.

In the second part of the study, participants were asked to imagine a similar attack by Russia occurring on French soil (condition *ingroup target*) and answered questions checking the level of detail and clarity in their imagined vision of the attack. The same measures of judgment of the Russian attack on France, judgment of Putin, and a feeling thermometer were completed again.^3^ Finally, participants filled in various socio-demographic information and were fully debriefed.

#### Measures

Unless otherwise indicated, the items were rated on a 7-point scale from 1: *Strongly disagree* to 7: *Strongly agree*. Judgments of the Russian attack (on outgroup or ingroup), Putin, and the Russian people were measured twice: once after the article about the Russian attack on Ukraine and once after participants imagined a Russian attack on France.

**National Narcissism** was measured with the 5-item short version of the collective narcissism scale (Golec de Zavala et al., 2009), referring to France. The five items were: 1. France deserves special treatment. 2. I will never be satisfied until France gets the recognition it deserves. 3. It makes me angry when people criticize France. 4. If France had a major say in the world, the world would be a much better place. 5. Not many people seem to fully understand the importance of France.

**National Satisfaction** was measured with the four items of the ingroup satisfaction sub-dimension from ingroup identification (Leach et al., 2008; see e.g., Golec de Zavala et al., 2020, for similar use of this sub-dimension), referring to French people. The four items were as follows: 1. I am glad to be French. 2. I think that French people have a lot to be proud of. 3. It is pleasant to be French. 4. Being French gives me a good feeling.

**Perception of Belief Similarity With Putin and With Russians** was measured with two items ‘How similar do you think your beliefs about and attachment to France are to President Vladimir Putin’s beliefs about and attachment to Russia?’ and ‘How similar do you think your beliefs about and attachment to France are to Russians’ beliefs about and attachment to Russia?’, rated on a 7-point scale from 1: *Very different* to 7: *Very similar*.

**Moral Judgment of Russian Attack.** Participants were asked to rate the extent to which the attack was ‘severe’ [reverse-coded], ‘justified’, ‘moral’, ‘legitimate’. The item ‘freely chosen’ contributed to a substantive decrease in the alpha and was removed. A higher score on the scale corresponds to a judgment of the attack on Ukraine or France as more moral and justified.

**Social Judgment of Putin** was measured with eight items (‘competent’, ‘intelligent’, ‘friendly’, ‘empathetic’, ‘fair’, ‘trustworthy’).^4^

**Judgment of Russians** was measured through a feeling thermometer. Participants were asked to evaluate their judgment of Russian people (among four other groups: French, Ukrainians, Europeans, Americans) using a slider scale ranging from 0 to 100.

**Check of the Clarity of the Imagined Attack on France** was measured by three items (adapted from de Place & Bruno, 2018), α = .77, *M* = 3.63, *SD* = 1.57. The three items were as follows: 1. When you imagine this situation, you have the impression that you’re seeing the images clearly in your mind. (Rated on a scale from 1: *Not at all* to 7: *Very much*) 2. How often do you think about this situation? (Rated on a scale from 1: *Never* to 7: *Very frequently*) 3. How likely do you think it is that the situation you imagined could happen? (rated on a scale from 1: *Not at all probable* to 7: *Very probable*).

Means and standard deviations of the variables can be found in Table 1. Correlations between variables can be found in Table 2. Notably, national narcissism is correlated with all dependent variables of interest while national satisfaction is only correlated with judgments of similarity and perceptions of the attack on the ingroup.

**Table 1:** Reliability and Descriptive Statistics of Focal Variables (Study 1).


	TOTAL		UKRAINE CONDITION		INGROUP CONDITION	
		
	α/*r*^1^	*M (SD)*	α	*M (SD)*	α	*M (SD)*

National narcissism	.89	3.02 (1.54)				

National satisfaction	.93	5.08 (1.58)				

Similarity	.59	2.81 (1.77)				

Judgment of the attack			.89	1.83 (1.35)	.84	1.59 (1.04)

Judgment of Putin			.88	2.54 (1.28)	.87	2.34 (1.25)

Judgment of Russians				42.61 (29.39)		32.98 (31.31)


*Note*: ^1^ The Pearson’s *r* was used for the similarity measure, as the scale contained only two items.

**Table 2:** Correlations Between the Focal Variables (Study 1).


VARIABLE	1	2	3	4	5	6	7	8	9

1. National narcissism	—	.55***	.37***	.21***	.04	.14*	.11*	–.09	–.17**

2. National satisfaction		—	.30***	–.07	–.18**	–.05	–.07	.04	–.09

3. Similarity			—	.37***	.23***	.46***	.43***	.25***	.12*

4. Judgment of the attack (Ukraine condition)				—	.72***	.81***	.77***	.29***	.27***

5. Judgment of the attack (ingroup condition)					—	.64***	.69***	.20***	.24***

6. Judgment of Putin (Ukraine condition)						—	.93***	.37***	.32***

7. Judgment of Putin (ingroup condition)							—	.37***	.37***

8. Judgment of Russians (Ukraine condition)								—	.80***

9. Judgment of Russians (ingroup condition)									—


* *p* < .05. ** *p* < .001. *** *p* < .001.

### Results

#### Effects of the Target and National Narcissism^5^

##### Moral Judgment of Russian Attack

**Figure 1:**
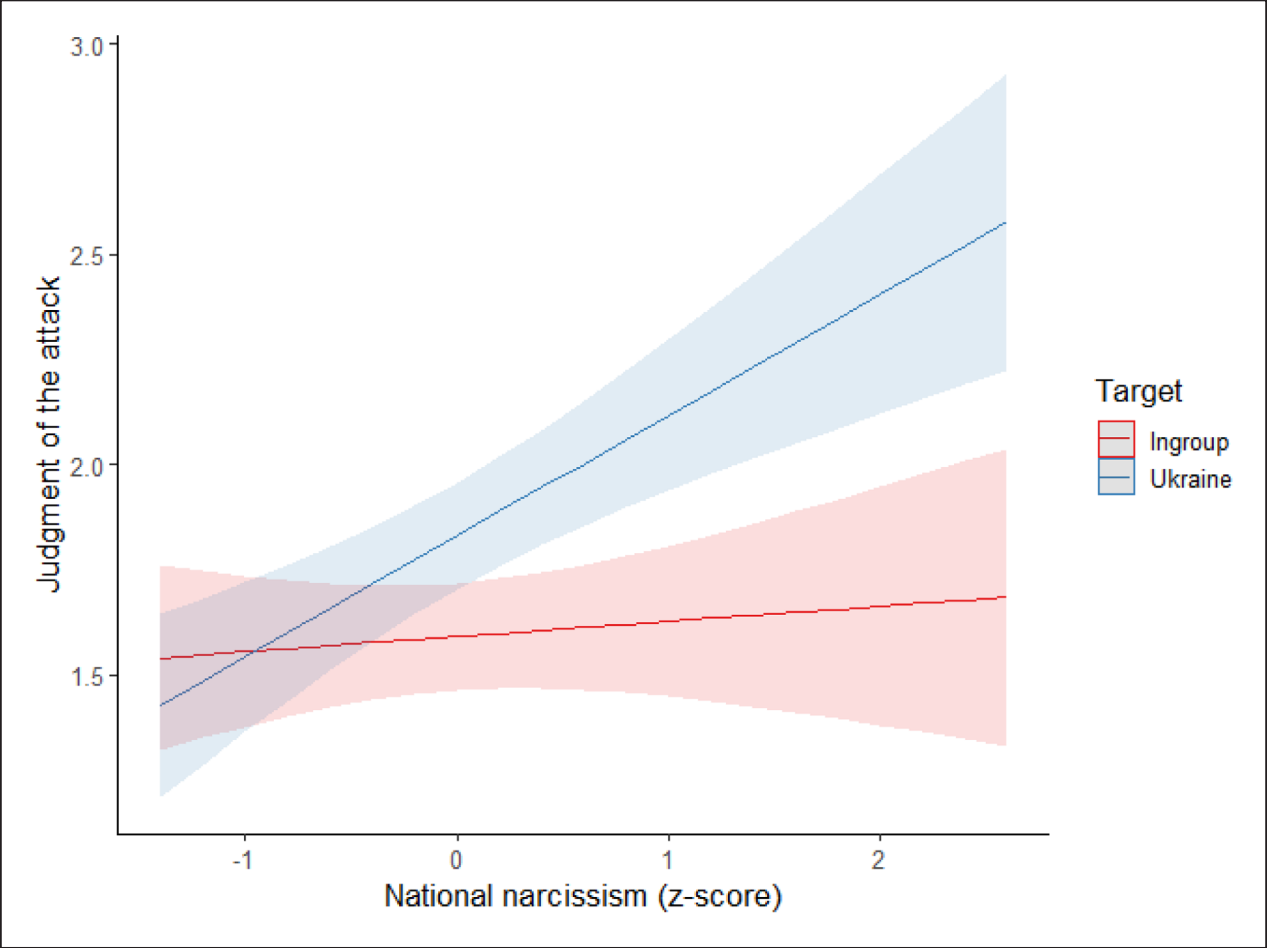
Association Between National Narcissism and Judgment of the Attack per Condition (Study 1).

We conducted a mixed model on the judgment of the Russian attack, with the target (ingroup vs. Ukraine; within-subjects), and national narcissism (*z*-score) as predictors, see Figure 1.

There was a main effect of the target, *F*(1,337) = 23.74, *p* < .001, η²_p_ = .07. Although the attack was judged as immoral in both conditions, the judgment of immorality was less severe in the Ukraine condition (estimated *M* = 1.83, *SD* = 0.07) than the ingroup condition (estimated *M* = 1.59, *SD* = 0.06). As expected, the interaction between national narcissism and target was significant, *F*(1,337) = 26.26, *p* < .001, η²_p_ = .07. Specifically, national narcissism was positively related to the moral judgment of the attack in the Ukraine, *B* = 0.28, *SE(B)* = 0.07, *t* = 4.02, *p* < .001, η²_p_ = .05, but not in the ingroup condition, *B* = 0.04, *SE(B)* = 0.06, *t* = 0.64, *p* = .525, η²_p_ = .001 (main effect of national narcissism: *F*(1,337) = 7.34, *p* = .007, η²_p_ = .02). While there was no difference in judgment among low national narcissists (at –1 *SD*; Ukraine target: *M* = 1.54, *SD* = 0.10; ingroup target: *M* = 1.56, *SD* = 0.08), *p* = .854, the attack on Ukraine was judged as more moral than the attack on the ingroup among higher national narcissists (at +1 *SD*; Ukraine target: *M* = 2.12, *SD* = 0.10; ingroup target: *M* = 1.63, *SD* = 0.08), *p* < .001.

##### Social Judgment of Putin

**Figure 2:**
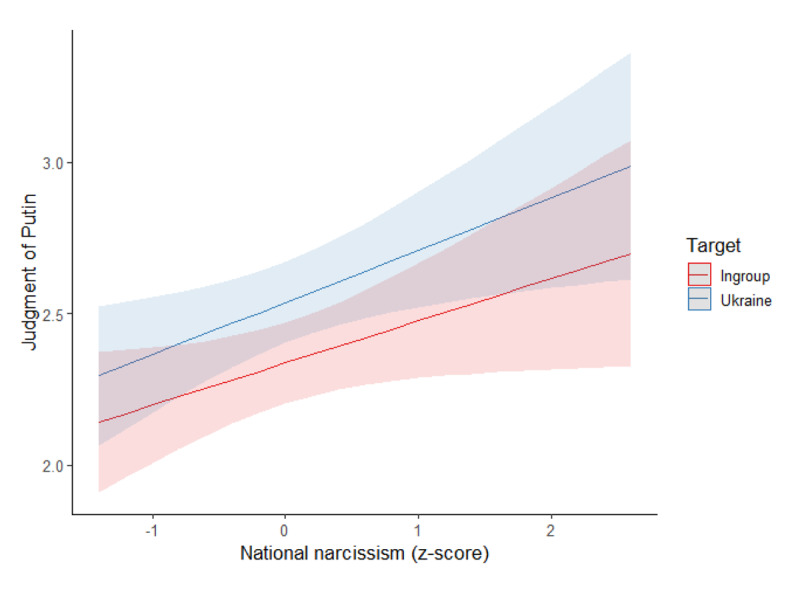
Association Between National Narcissism and Judgment of Putin per Condition (Study 1).

We conducted the same model on the judgment of Putin, see Figure 2. The target had a main effect, *F*(1,337) = 63.20, p < .001, η²_p_ = .16, so that Putin was judged more positively in the Ukraine (estimated *M* = 2.54, *SD* = 0.07) than in the ingroup attack condition (estimated *M* = 2.34, *SD* = 0.07). National narcissism had a positive main effect, *F*(1,337) = 5.41, *p* = .021, η²_p_ = .02, on the judgment of Putin. The interaction of the target with national narcissism was not significant, *F*(1,337) = 1.82, *p* = .178, η²_p_ = .01.

##### Judgment of Russians

**Figure 3:**
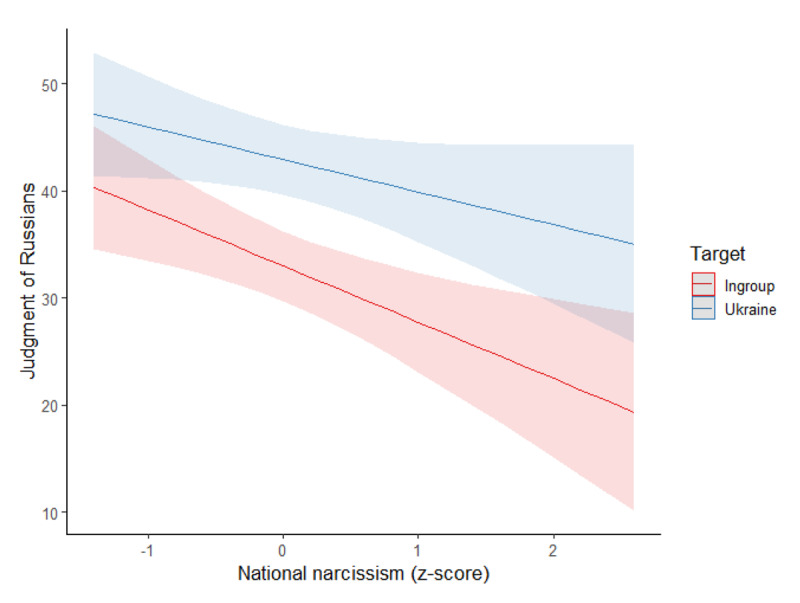
Association Between National Narcissism and Judgment of Russians per Condition (Study 1).

The same model was conducted on the judgment of Russians, see Figure 3. Again, we found a main effect of the target, *F*(1,319) = 87.76, *p* < .001, η²_p_ = .22, with Russians being judged more positively when the target of the attack was Ukraine (estimated *M* = 42.87, *SD* = 1.62) than when it was the ingroup (estimated *M* = 32.88, *SD* = 1.72). Moreover, the interaction of the target with national narcissism was significant, *F*(1,319) = 4.16, *p* = .042, η²_p_ = .01. Specifically, national narcissism was related to a more negative judgment of Russians when the target was the ingroup, *B* = –5.24, *SE(B)* = 1.75, *t* = –2.99, *p* = .003, η²_p_ = .03, but not when it was Ukraine, *B* = –3.04, *SE(B)* = 1.65, *t* = –1.84, *p* = .067, η²_p_ = .01 (main effect of national narcissism: *F*(1,319) = 6.58, *p* = .011, η²_p_ = .02). At lower levels of national narcissism (–1 *SD*), Russians were judged more positively in the Ukraine condition (*M* = 45.97, *SD* = 2.34) than in the ingroup condition (*M* = 32.23, *SD* = 2.48), *p* < .001. The same pattern was observed at elevated levels of national narcissism (+1 *SD*; Ukraine target: *M* = 39.90, *SD* = 2.29; ingroup target: *M* = 27.75, *SD* = 2.43).

#### Mediation Through Perceived Similarity

To test the potential mediation through similarity, we conducted a structural equation model (SEM) analysis using R package Lavaan (Rosseel, 2012). We used latent variables, considering national narcissism as the predictor, similarity as the mediator, and the judgment of the Russian attack (Model 1) and of Putin (Model 2) in the Ukraine and ingroup conditions as two parallel outcomes (complete results of the SEM analyses can be found as Supplemental Material). We applied a 5,000 resample bootstrap. Since we did not find the predicted positive association between national narcissism and judgment of Russians, we do not present the mediation analysis on judgment of Russians here. We do, however, include it in Supplemental Material.

##### Moral Judgment of Russian Attack

**Figure 4:**
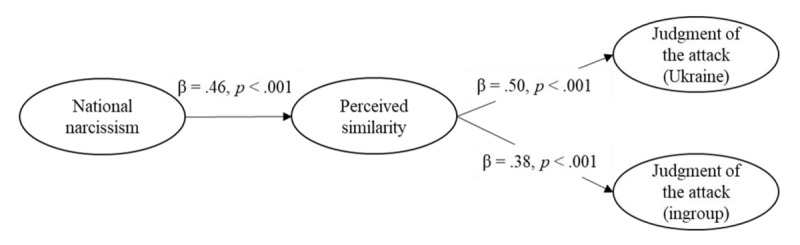
Structural Equation Model on the Judgment of the Attack (Study 1).

Model 1 had an acceptable fit, χ²(80) = 163.20, *p* < .001, CFI = .973, RMSEA = 0.056, 90%CI [0.044, 0.068], SRMR = 0.039. This model revealed that national narcissism was positively associated with perceived similarity with Putin and Russians (see Figure 4). In turn, similarity was associated with a less negative judgment of the attack both in the Ukraine and the ingroup condition. Moreover, the indirect effects from national narcissism to judgment of the attack through perceived similarity was significant in Ukraine, *B* = 0.24, *SE*(*B*) = 0.06, 95% CI [0.13, 0.38], *z* = 3.85, *p* < .001, and ingroup conditions,^6^ *B* = 0.15, *SE*(*B*) = 0.04, 95% CI [0.08, 0.24], *z* = 3.62, *p* < .001. The contrast indicated that the two indirect effects differed, *B* = 0.09, *SE*(*B*) = 0.04, 95% CI [0.03, 0.17], *z* = 2.54, *p* = .011, although both were significant. In other words, the more participants expressed national narcissism towards their country, the more they were prone to perceive themselves as having similar beliefs to Putin and Russians, which in turn was associated with the judgment of an attack (either the Ukrainian outgroup or, to a lesser extent, the ingroup) as less immoral.

##### Social Judgment of Putin

**Figure 5:**
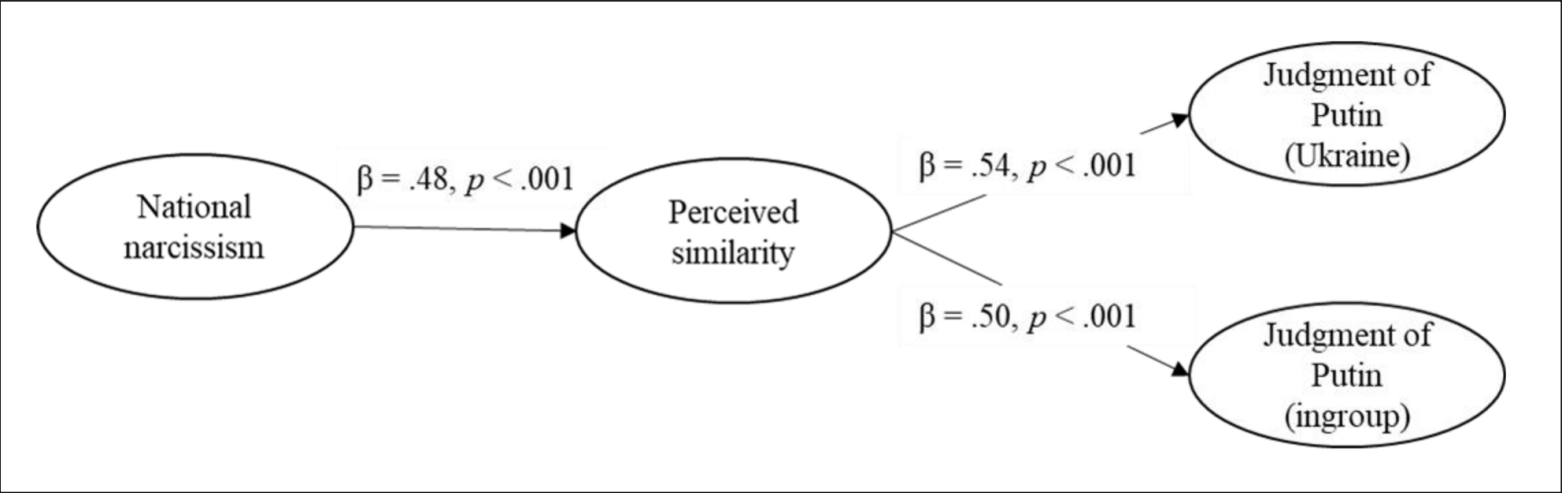
Structural Equation Model on the Judgment of Putin (Study 1).

Model 2 had an acceptable fit, χ²(140) = 452.69, *p* < .001, CFI = .945, RMSEA = 0.082, 90%CI [0.074, 0.091], SRMR = 0.068. National narcissism was positively related to perceived similarity (see Figure 5). Higher perceived similarity was related to a less negative judgment of Putin both in the Ukraine and ingroup conditions. The indirect effects from national narcissism to judgment of Putin through perceived similarity were significant for the Ukraine, *B* = 0.24, *SE*(*B*) = 0.06, 95% CI [0.14, 0.36], *z* = 4.19, *p* < .001, and ingroup condition, *B* = 0.23, *SE*(*B*) = 0.06, 95% CI [0.13, 0.35], *z* = 4.07, *p* < .001, and did not differ between the conditions, *p* = .419. In sum, higher national narcissists were more inclined to perceive similarity with Putin and Russians, and thus to judge Putin more favorably regardless of whether the attack was on Ukraine or the ingroup.

### Discussion of Study 1

Study 1 provided support for our hypothesis that people with stronger national narcissistic beliefs are more inclined to judge the Russian attack on Ukraine as less immoral. Consistent with our hypotheses, this was moderated by the target as there was no relation between national narcissism and moral judgment of a potential attack on France, the ingroup. Still, we did observe in both cases an indirect effect through perceived belief similarity. However, national narcissism was related to a more positive judgment of Putin regardless of whether the attack they read about or imagined was on an outgroup or on the ingroup. Again, these effects were explained by a higher perceived belief similarity with Putin and Russians on the part of those higher in national narcissism. However, this positive judgment did not extend to Russian people, who were judged more negatively by higher national narcissists in the ingroup condition, while there was no link in the Ukraine condition.

## Study 2

Study 2 replicates Study 1 but on an American population, i.e., in a context in which the threat is less direct but with an intense history of political conflict with Russia. As in Study 1, the main hypotheses, namely the link between national narcissism and less immoral judgment of military attack, less negative judgment of Putin, and the Russian people, particularly when the attack is on an outgroup (Ukraine) rather than the ingroup (US) were pre-registered (https://osf.io/d8ku6). We also tested the potential mediating effect of perceived similarity (pre-registered as an exploratory model).

### Method

#### Participants

We recruited 413 participants online through Prolific (*n* = 216) and social media (Facebook, Twitter; *n* = 197) from 9 March to 12 May 2022. The questionnaire was shared in groups related to politics (e.g., ‘US Politics’, ‘Washington DC Young Republicans’, ‘Democrats for Biden’) and groups with individual states’ names in the title (e.g., ‘For Sale by Owner – North Carolina’). We excluded participants who were not of US nationality (*n* = 7), who did not answer both attention checks correctly (*n* = 2) and who failed to imagine a Russian attack on the US (*n* = 8). The final sample consisted of 400 participants (155 men, 226 women, 19 non-binary), aged 18 to 91 (*M* = 36.03, *SD* = 13.99). Our sample was more left-wing leaning than right-wing (*M* = 2.80, *SD* = 1.76).

#### Procedure and Measures

The procedure and measures were strictly identical to Study 1, except that the measures and experimental induction mentioning the ingroup (e.g., national narcissism) referred to the US ingroup.

Reliability indexes and descriptive statistics can be found in Table 3, and correlations in Table 4.

**Table 3:** Reliability and Descriptive Statistics of Focal Variables (Study 2).


	TOTAL		UKRAINE CONDITION		INGROUP CONDITION	
		
	α/*r*^1^	*M (SD)*	α	*M (SD)*	α	*M (SD)*

National narcissism	.89	2.43 (1.28)				

National identification	.95	4.39 (1.66)				

Similarity	.51	2.97 (1.50)				

Judgment of the attack			.88	1.37 (0.77)	.75	1.40 (0.71)

Judgment of Putin			.81	2.27 (0.88)	.72	1.40 (0.71)

Judgment of Russians				44.18 (23.70)		31.14 (26.16)


*Note*: ^1^ The Pearson’s *r* was used for the similarity measure, as the scale contained only two items.

**Table 4:** Correlations Between the Focal Variables (Study 2).


VARIABLE	1	2	3	4	5	6	7	8	9

1. National narcissism	—	.62***	.34***	.20***	.16**	.21***	.19***	–.02	–.08

2. National satisfaction		—	.41***	.13**	.003	.24***	.26***	.07	.01

3. Similarity			—	.25***	.20***	.38***	.36***	.16**	.09

4. Judgment of the attack (Ukraine condition)				—	.55***	.61***	.47***	.13**	.15**

5. Judgment of the attack (ingroup condition)					—	.37***	.38***	.03	.12*

6. Judgment of Putin (Ukraine condition)						—	.78***	.26***	.23***

7. Judgment of Putin (ingroup condition)							—	.20***	.22***

8. Judgment of Russians (Ukraine condition)								—	.77***

9. Judgment of Russians (ingroup condition)									—


* *p* < .05. ** *p* < .001. *** *p* < .001.

### Results

#### Effects of the Target and National Narcissism

##### Moral Judgment of Russian Attack

**Figure 6:**
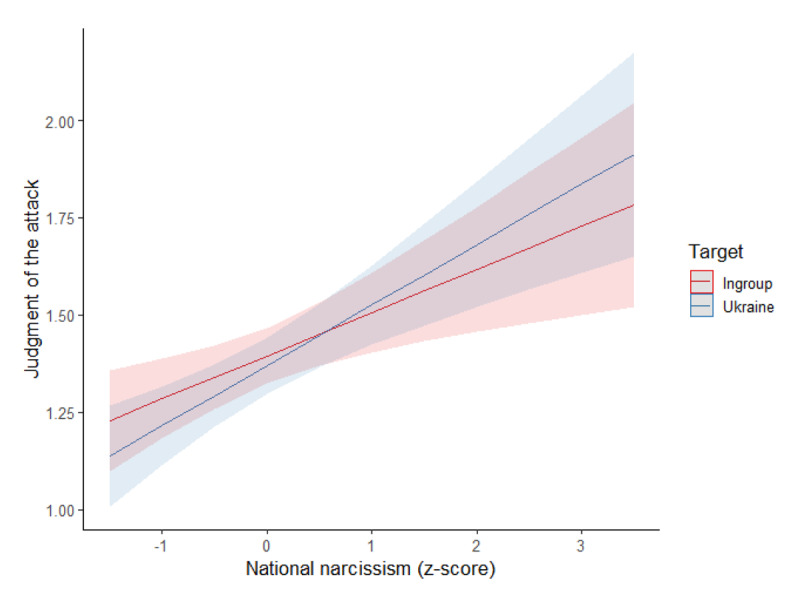
Association Between National Narcissism and Judgment of the Attack per Condition (Study 2).

We conducted a mixed model on the judgment of the Russian attack, with the target (ingroup vs. Ukraine; within-subjects), and national narcissism (*z*-score) as predictors, see Figure 6.

The target had no main effect on the judgment of the attack, *F*(1,398) = 0.50, *p* = .481, η²_p_ = .001. National narcissism was related to a less immoral judgment of the attack, regardless of the condition, *F*(1,398) = 17.12, *p* < .001, η²_p_ = .04 (interaction national narcissism × target: *F*(1,398) = 1.57, *p* = .211, η²_p_ = .004).

##### Social Judgment of Putin

**Figure 7:**
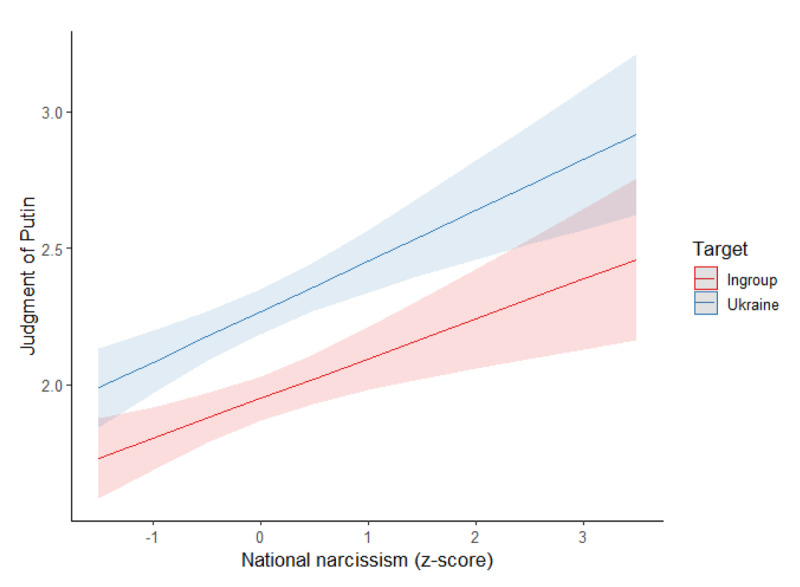
Association Between National Narcissism and Judgment of Putin per Condition (Study 2).

We conducted the same model on the social judgment of Putin, see Figure 7. The target had a main effect, *F*(1,395) = 125.36, *p* < .001, η²_p_ = .24, with Putin being judged more negatively when the target was the ingroup (estimated *M* = 2.27, *SD* = 0.04) than Ukraine (estimated *M* = 1.95, *SD* = 0.04). National narcissism was associated with a better judgment of Putin, *F*(1,395) = 18.69, *p* < .001, η²_p_ = .05, and this was not moderated by the target, *F*(1,395) = 2.02, *p* = .156, η²_p_ = .01.

##### Judgment of Russians

**Figure 8:**
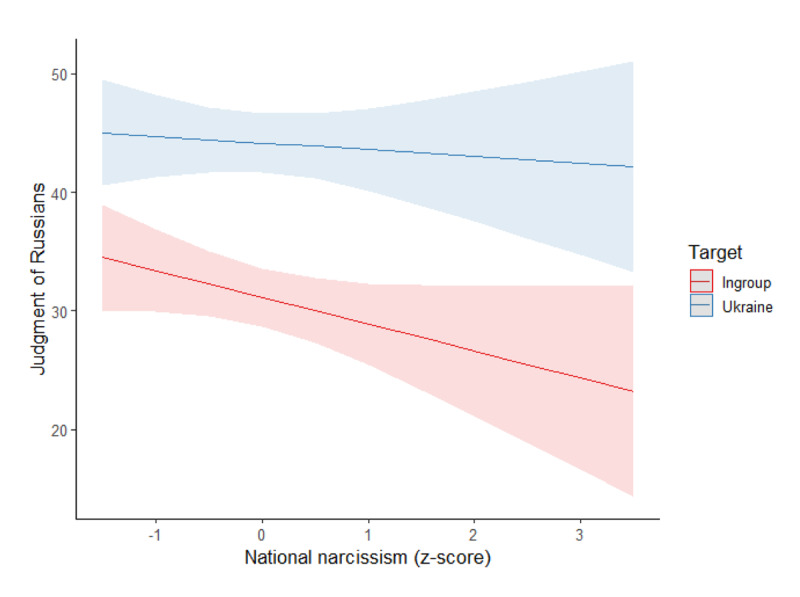
Association Between National Narcissism and Judgment of Russians per Condition (Study 2).

Finally, we conducted the same analyses on the judgment of Russians, see Figure 8. The target had a main effect, *F*(1,392) = 226.11, *p* < .001, η²_p_ = .37, with a more positive judgment of Russians in the Ukraine target condition (estimated *M* = 44.22, *SD* = 1.20) than in the ingroup condition (estimated *M* = 31.15, *SD* = 1.32).

National narcissism, *F*(1,392) = 1.12, *p* = .291, η²_p_ = .003, was not related to the judgment of Russians, and this was not moderated by the target, *F*(1,392) = 3.67, *p* = .056, η²_p_ = .01.

#### Mediation Through Perceived Similarity

We conducted similar analyses to Study 1 to examine indirect effects through perceived similarity, i.e., SEM with national narcissism as predictor, similarity as mediator, and judgment of the attack and of Putin in Ukraine and ingroup conditions as parallel outcomes, with 5,000 bootstraps. As in Study 1, we do not present the mediation analysis on judgment of Russians since national narcissism was not related with this dependent variable, but the analysis is included in Supplemental Material.

##### Moral Judgment of Russian Attack

**Figure 9:**
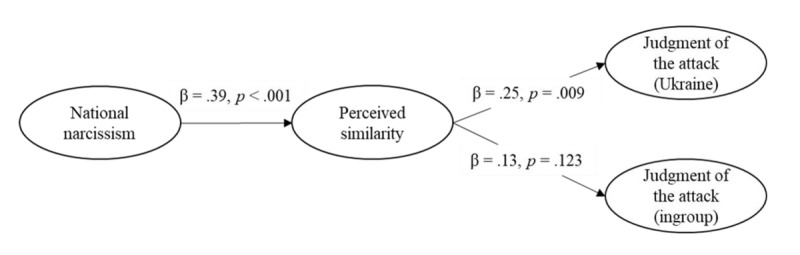
Structural Equation Model on the Judgment of the Attack (Study 2).

The model conducted on the judgment of the attack had an adequate fit, χ²(80) = 185.96, *p* < .001, CFI = .969, RMSEA = 0.058, 90%CI [0.047, 0.068], SRMR = 0.044. National narcissism was positively associated with perceived similarity. Perceived similarity was positively associated with the judgment of the attack in the Ukraine but not in the ingroup condition (see Figure 9).

Indirect effects from national narcissism to judgment of the attack through similarity were significant in the Ukraine, *B* = 0.08, *SE*(*B*) = 0.03, 95% CI [0.02, 0.15], *z* = 2.35, *p* = .019, but not in the ingroup condition, *B* = 0.04, *SE*(*B*) = 0.02, 95% CI [0.00, 0.09], *z* = 1.46, *p* = .143, and these two indirect effects differed, *B* = 0.04, *SE*(*B*) = 0.02, 95% CI [0.01, 0.08], *z* = 2.16, *p* = .031.

##### Social Judgment of Putin

**Figure 10:**
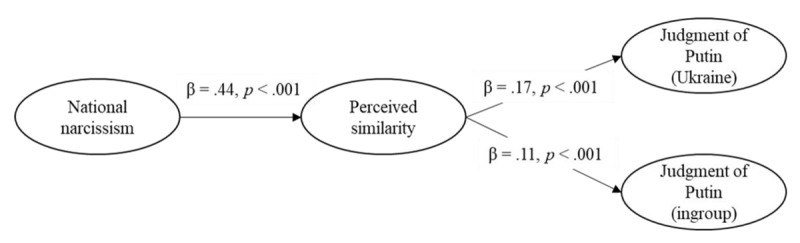
Structural Equation Model on the Judgment of Putin (Study 2).

The same SEM conducted on the judgment of Putin had a weak fit, χ²(140) = 715.61, *p* < .001, CFI = .886, RMSEA = 0.102, 90%CI [0.094, 0.109], SRMR = 0.089, and results should therefore be considered with caution. National narcissism was positively associated with perceived similarity. Similarity was positively associated with the judgment of Putin both in the Ukraine and ingroup conditions (see Figure 10).

Indirect effects from national narcissism to judgment of Putin through similarity was significant in the Ukraine, *B* = 0.11, *SE*(*B*) = 0.03, 95% CI [0.05, 0.18], *z* = 3.33, *p* = .001, and ingroup condition, *B* = 0.07, *SE*(*B*) = 0.03, 95% CI [0.02, 0.13], *z* = 2.46, *p* = .014. These effects differed, *B* = 0.04, *SE*(*B*) = 0.01, 95% CI [0.01, 0.07], *z* = 2.80, *p* = .005, indicating that the indirect effect was stronger in the Ukraine than in the ingroup condition. In sum, people who expressed more national narcissism were more inclined to perceive belief similarity with Putin and Russians, which in turn was associated with a less negative judgment of Putin. These indirect effects were found both when the attack was on the ingroup or on Ukraine, although it was stronger in this latter case.

### Discussion of Study 2

Study 2’s results are consistent with Study 1. In both studies, national narcissism was related to a judgment of the attack of Ukraine as less immoral. In Study 2 however, national narcissism was related to the judgment of the attack as less immoral regardless of the target (albeit the indirect effect through perceived similarity held only for the attack on Ukraine). As in Study 1, Putin was judged more positively by higher national narcissists, regardless of the target and this was partly explained by perceived similarity, especially in the Ukraine condition. Finally, Study 2 also suggested that these more positive evaluations do not extend to Russians as national narcissism was not associated with judgment of Russians.

## Discussion

In this pair of studies, we found support for our hypothesis that levels of national narcissism and perceived belief similarity with Russia and Putin could influence how people around the world view the conflict between Ukraine and Russia. We made the general hypothesis that national narcissism could be related to support for outgroups’ nationalist leaders and actions, even if this ultimately represents a threat to the ingroup. We thus relied on the context of intergroup violence and conflict between Russia and Ukraine which began in February 2022 to test this hypothesis. We also hypothesized that the explicit target of the attack (Ukraine vs. ingroup) could alleviate support for intergroup violence and Putin among high national narcissists. Finally, we examined the potential mediating role of perceived belief similarity.

Study 1, in France, and 2, in the US, partly confirmed our general hypothesis, showing that national narcissism was related to the perception of an attack as less immoral, especially when it targeted Ukraine. In France, no association was observed between national narcissism and the judgment of the attack on France. However, the indirect effects through perceived similarity were still significant for both targets. In the US, national narcissism was related to the judgment of the attack targeting either Ukraine or the US as less immoral, but this was not driven by perceived similarity when targeting the ingroup. Additionally, in both studies, those higher in national narcissism judged Putin less negatively, regardless of the target. In both studies, this better judgment of Putin from higher national narcissists was partly explained by a higher perceived belief similarity. Finally, those more positive feelings did not extend to the Russian people. National narcissism was mostly unrelated to opinions about the Russian people except in France where we observed a negative association when a Russian attack on France was imagined.

### National Narcissism, Belief Similarity and Support for Outgroup Nationalist Leaders and Intergroup Violence

In our research, we found a consistent link between national narcissism and support for military attacks, in two distinct cultural and relational contexts with Russia. Moreover, in both contexts, we observed indirect effects of national narcissism via belief similarity, leading to less immoral judgment of military attacks and a better judgment of Putin. It seems, therefore, that people with higher levels of national narcissism perceive themselves as similar in identity to Russians and Putin, i.e., to a nationalist leader. This perceived belief similarity leads, in both contexts, to more support for the outgroup leader and his actions.

Many studies have shown that national narcissism is linked to more support for ingroup nationalist leaders and policies on the one hand (Golec de Zavala & Keenan, 2021), and to hostility towards threatening outgroups on the other (Cichocka & Cislak, 2020). The present research provides complement to this literature by showing that national narcissism can be linked to more support for nationalist actions and leaders of an *outgroup*, even when the outgroup poses a direct threat to the ingroup. Indeed, our study highlights the importance of perceived belief similarity in both the Ukraine and ingroup conditions. Just as individuals value others who are like them (Bocian et al., 2018), individuals with higher levels of national narcissism more positively evaluated the actions of people perceived as sharing national narcissist beliefs.

These results are in line with recent work suggesting that collective narcissism may have deleterious effects on the group itself (Cichocka et al., 2022; Gronfeldt et al., 2022; Gronfeldt, et al., 2023). A member of the outgroup may be de facto threatening, but at the same time be considered ideologically close and thus receive more positive attitudes. Thus, we call for consideration of the beliefs dimension of national narcissism, in supplement to the intergroup dimension, when considering consequences on intergroup attitudes. Our results suggest that national narcissists may prefer to defend their way of thinking rather than their group and its members.

This may be because they identify with a superordinate group which includes people of diverse nations who all hold national narcissistic beliefs, similar to other groups with shared beliefs like a religion or a global environmental group. This judgment of similarity may be what drives their actions. Alternatively, national narcissists may see groups of national narcissists from other countries as a means to promote their own nationalist strategies and prove their value. Although, our study cannot shed light on which of these processes may be influencing national narcissists’ support of the war, it is clear that, in line with other studies (Cichocka et al., 2022; Gronfeldt et al., 2022), we show that national narcissists’ decisions (i.e., condoning a threatening nationalist leader) are not always in the best interest of their group.

### Limitations and Future Directions

Our research provides first insights in understanding identity processes related to support for outgroup nationalism. Yet, several limitations should be noted. First, our reliance on cross-sectional data does not allow us to establish a clear causal relation between identity-related beliefs and support for outgroup populist leaders and actions. Also, our data were collected early in the conflict. This conflict constantly evolves, with peaks of violence and threats (e.g., attack on nuclear power plant). Longitudinal data on national narcissism, perceptions of similarity, and evaluations of the conflict would increase understanding of the role of national narcissism in supporting nationalist regimes over time.

Second, we observed only a few effects based on whether the attack was on the ingroup or outgroup target, particularly in the US. We interpreted this as support for the ideological nationalist stance, regardless of the target of the attack, on the part of national narcissists. However, this may also be a sign that people failed to concretely imagine the situation as real. In fact, reactions to targets of the attack (ingroup vs. outgroup) did not differ in the US (where the threat of an actual attack was more unlikely) but we found some differences in France (where an actual threat had been formulated by Putin’s regime). Despite the high levels of anxiety at the beginning of the conflict (Le Monde, 2022) and our manipulation check, these results should be interpreted with caution and could be subject to change if an actual ingroup attack happened.

One interesting difference between the two countries merits further examination, namely, national satisfaction seemed to have similar associations to national narcissism with the judgment of the attack and Putin in the US (see also Supplemental Material). This could be due to the US’ longstanding tradition of exceptionalism (Gilmore & Rowling, 2018; Hartig, 2021) which may imply that a mere identification with the US relies on a grandiose form of pride. In any case, teasing apart how the concepts of national narcissism and national satisfaction differ in the US versus France could be an interesting avenue for future research.

Finally, while we interpreted our results as evidence that national narcissists support Putin because of their common beliefs, there could be alternative explanations. For example, rather than similarity in beliefs, perhaps seeing Putin as an ally against a common enemy may play a role in national narcissists support for the attack on Ukraine and Putin’s leadership. For instance, in France (and Europe more broadly) politicians have decried the perils of American hegemony on the world stage of politics. Thus, French national narcissists may support Putin because they see him as attempting to defeat American hegemony. While this explanation would not be applicable to our American participants, American national narcissists may see Putin as fighting the common enemy of liberal policies (e.g., rights for LGBTQ+ people) that they are less likely to agree with (Federico & Golec de Zavala, 2018).

## Conclusion

Our study is novel in that it is one of the first studies to examine political consequences of national narcissism on attitudes toward an *outgroup* political leader and a conflict between two outgroups. Previous work has explored various diasporas’ views of conflicts in relation to their ethnic, religious, or national identity (e.g., American Jew’s perceptions of Israel-Palestine Conflict, Hagai, et al, 2013; Irish-American responses to the Northern Ireland Conflict, Dochartaigh, 1995), but to our knowledge we are the first to examine attitudes toward outgroup conflicts between parties unrelated to the ingroup. Furthermore, we highlight the importance of accounting for similarity in beliefs across trans-national and trans-political groups in understanding the relationships between them. We also highlight that national narcissists may support actions which could harm their ingroup. In conclusion, national narcissism’s consequences go beyond the defense of one’s national ingroup and thus can result in important consequences for worldwide politics.

## Data Accessibility Statement

Data are available at https://osf.io/unkxy/.

## Additional File

The additional file for this article can be found as follows:

10.5334/irsp.761.s1 Supplementary file 1. Supplemental Material—Complete and complementary analyses.

## Footnotes

In this manuscript, we use nationalism as defined by Kosterman and Feshbach (1989), the perception that one’s nation is superior to others and therefore should dominate over others (see also Cichocka & Cislak, 2020). Therefore, we do not focus on banal nationalism or national identity as a community feeling but rather on ‘hot’, aggressive nationalism (Anderson, 1983; Billig, 1995; Kosterman & Feshbach, 1989) in the context of hostile intergroup relations. We therefore acknowledge that other theories of nationalism as an ideology, belief and process have been developed (Anderson, 1983; Billig, 1995; Reicher & Hopkins, 2000) but choose to use the term according to Kosterman and Feshbach’s (1989) definition.

Political orientation was measured with four items, asking participants to what extent do they feel close to the right on the one hand and the left on the other hand for both social and economic issues.

A measure of emotions was also present. As it is not in the scope of the present paper, we do not report or analyze it here.

Two items measuring assertiveness (‘self-confident’; ‘ambitious’) were also included. The exploratory factor analysis revealed two distinct dimensions: assertiveness vs. other dimensions of social judgment. Therefore, we chose to separate them and use only the general social judgment. Analyses on assertiveness can be found in Supplemental Material.

The focus on the article being on national narcissism, and due to high correlation with national satisfaction (especially in Study 2), we report the analyses considering solely national narcissism and target in the main manuscript. However, the pre-registered analyses including national satisfaction are reported in Supplemental Material.

Note that the total effect of national narcissism on the judgment of the attack on the ingroup is not significant, see Table 2, and this indirect effect should therefore be treated with caution.

## Open Access Declaration

For the purpose of Open Access, the authors have applied a Creative Commons Attribution (CC-BY) License to an Author Accepted Manuscript version arising.
