# Supplementary material for: We’re All the Same: Collective Narcissists’ Cross-National Support for Putin and Russian Military Attacks
Source: Int Rev Soc Psychol. 2024 Jan 17;37:1. doi: 10.5334/irsp.761 (PMC12372699; doi:10.5334/irsp.761)
Supplement: Supplementary file 1. — Supplemental Material—Complete and complementary analyses. [file irsp-37-761-s1.pdf]

Supplemental Material

Procedure Details (Study 1)

Ukraine Target Condition

Participants were reminded of Russia’s military action in Ukraine via this paragraph from Agence France Presse, “Shortly before 3am GMT on the night of Wednesday 23 February 2022, Russian President Vladimir Putin appeared on Russian television and announced that he had 'taken the decision on a military operation' in Ukraine, officially to defend Russian separatists. In the aftermath, bombing raids hit the whole of Ukraine, including the capital Kiev. Russian ground troops also entered the country.”

Ingroup (France) Target Condition

Participants were asked to imagine a similar attack on French soil using the following paragraph, “Now we would like you to take a few seconds to imagine an attack like the one in Ukraine happening in your country, France. During the night, the Russian president, Vladimir Putin announces an attack on your country. Russian planes bomb major cities in France and the Russian army arrives on French soil."

Complete Results of the Structural Equation Model Analyses (Study 1)

On Moral Judgment of the Attack

Figure S1

Tested Structural Equation Model on Judgment of the Attack

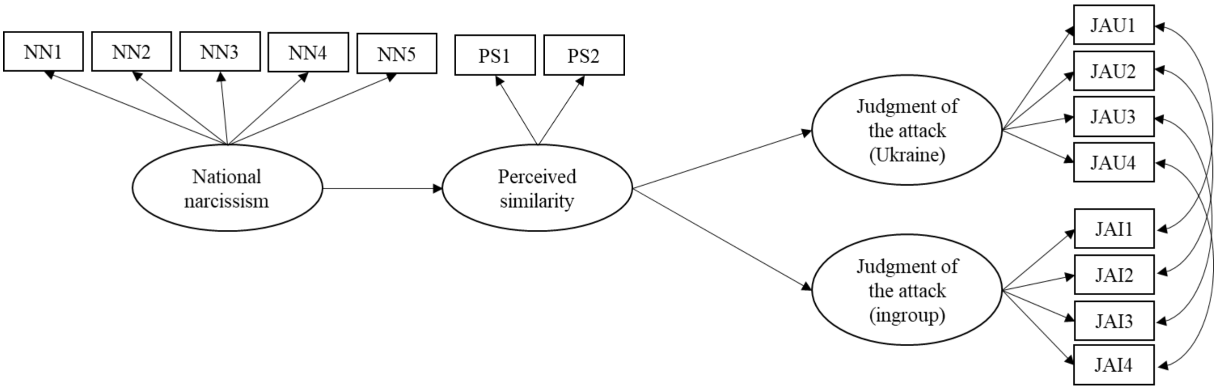

Table S1

Loadings of Items on Latent Variables

Variable/Item	*B*	*SE(B)*	*z*	*p*	95%CI		β
Judgment of the attack—Ukraine condition							
Item 1 (justified)	1.00				1.00	1.00	.89
Item 2 (moral)	0.67	0.06	12.18	.000	0.56	0.78	.78
Item 3 (legitimate)	1.00	0.04	25.96	.000	0.93	1.08	.95
Item 4 (severe)	-0.62	0.06	-10.14	.000	-0.74	-0.50	-.66
Judgment of the attack—Ingroup condition							
Item 1 (justified)	1.00				1.00	1.00	.87
Item 2 (moral)	0.61	0.07	9.05	.000	0.48	0.74	.77
Item 3 (legitimate)	0.97	0.05	20.38	.000	0.89	1.08	.96
Item 4 (severe)	-0.46	0.09	-5.10	.000	-0.65	-0.29	-.48
National narcissism							
Item 1 (France deserves special treatment)	1.00				1.00	1.00	.74
Item 2 (I will never be satisfied until France gets the recognition it deserves)	1.23	0.08	15.06	.000	1.09	1.41	.85
Item 3 (It makes me angry when people criticize France)	1.11	0.11	10.39	.000	0.92	1.33	.73
Item 4 (If France had a major say in the world, the world would be a much better place)	1.07	0.09	11.44	.000	0.91	1.28	.78
Item 5 (Not many people seem to fully understand the importance of	1.10	0.10	11.38	.000	0.93	1.30	.80

France)

			Perceived similarity				
Item 1 (Similarity with Putin)	1.00				1.00	1.00	.97
Item 2 (Similarity with Russians)	0.69	0.09	7.39	.000	0.51	0.88	.60

Table S2

Regressions Analyses

Variable	*B*	*SE(B)*	*z*	*p*	95%CI		β
Judgment of the attack—Ukraine condition (R^2 = .23)							
National narcissism	0.08	0.08	0.92	.358	-0.09	0.25	.06
Perceived similarity	0.38	0.08	4.60	.000	0.23	0.55	.44
Judgment of the attack—Ingroup condition (R^2 = .09)							
National narcissism	-0.07	0.06	-1.12	.263	-0.19	0.05	-.07
Perceived similarity	0.23	0.05	4.35	.000	0.13	0.35	.33
Perceived similarity (R^2 = .21)							
National narcissism	0.64	0.10	6.49	.000	0.45	0.83	.46

Table S3

Indirect and Total Effects of the Mediation Analysis on Judgement of the Attack in French Sample

Effect	*B*	*SE(B)*	*z*	*p*	95%CI		β
Indirect effects							
National narcissism → Similarity → Judgment of the attack—Ukraine condition (Indirect NN U)	0.24	0.06	3.85	.000	0.13	0.38	0.20
National narcissism → Similarity → Judgment of the attack—Ingroup condition (Indirect NN I)	0.15	0.04	3.62	.000	0.08	0.24	0.15
Contrast 1: Indirect NN U - Indirect NN I	0.09	0.04	2.54	.011	0.03	0.17	0.05
Total effects							
National narcissism → Judgment of the attack—Ukraine condition	0.32	0.09	3.69	.000	0.15	0.49	0.27
National narcissism → Judgment of the attack—Ingroup condition	0.08	0.06	1.33	.184	-0.04	0.20	0.08

On Social Judgment of Putin

Figure S2

Tested Structural Equation Model on Judgment of Putin

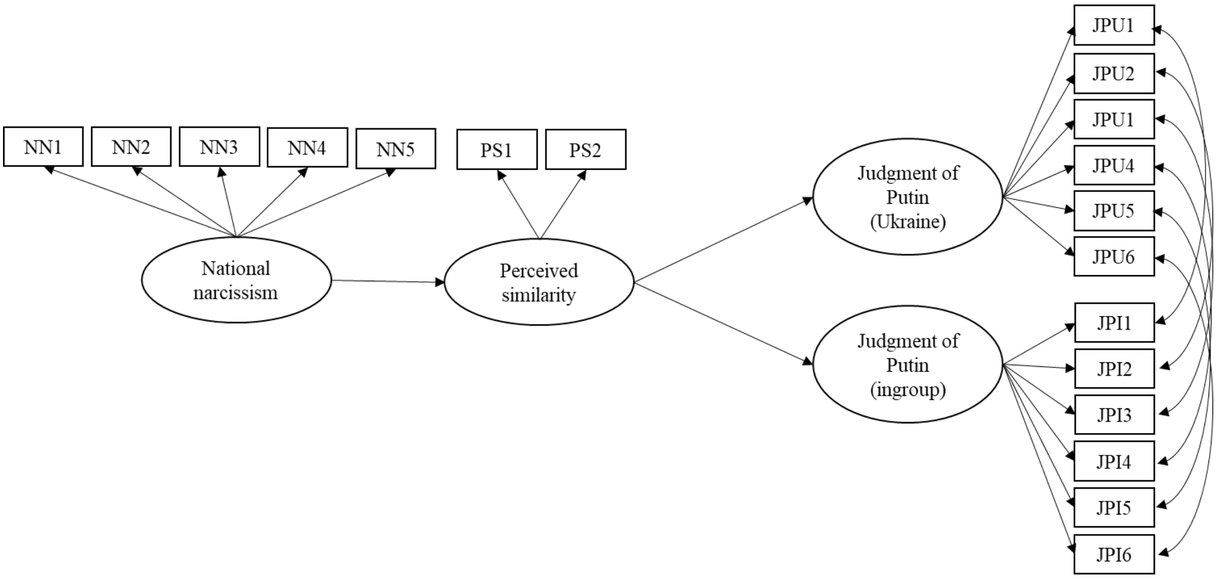

Table S4

Loadings of Items on Latent Variables

Variable/Item	*B*	*SE(B)*	*z*	*p*	95%CI		β
Judgment of Putin—Ukraine condition							
Item 1 (competent)	1.00				1.00	1.00	.62
Item 2 (intelligent)	0.71	0.06	12.18	.000	0.60	0.83	.45
Item 3 (friendly)	0.99	0.08	12.76	.000	0.86	1.16	.89
Item 4 (empathetic)	0.87	0.09	10.09	.000	0.72	1.05	.79
Item 5 (fair)	1.03	0.08	12.68	.000	0.89	1.21	.89
Item 6 (trustworthy)	1.14	0.09	13.18	.000	0.99	1.32	.87
Judgment of Putin—Ingroup condition							
Item 1 (competent)	1.00				1.00	1.00	.60
Item 2 (intelligent)	0.77	0.05	15.99	.000	0.67	0.86	.45
Item 3 (friendly)	0.96	0.08	12.74	.000	0.82	1.12	.92
Item 4 (empathetic)	0.82	0.07	11.33	.000	0.68	0.96	.80
Item 5 (fair)	0.95	0.09	10.72	.000	0.78	1.12	.86
Item 6 (trustworthy)	1.13	0.09	13.31	.000	0.98	1.31	.90
National narcissism							
Item 1 (France deserves special treatment)	1.00				1.00	1.00	.73
Item 2 (I will never be satisfied until	1.25	0.09	14.60	.000	1.11	1.44	.84

France gets the recognition it deserves)							
Item 3 (It makes me angry when people criticize France)	1.13	0.12	9.85	.000	0.92	1.38	.73
Item 4 (If France had a major say in the world, the world would be a much better place)	1.09	0.10	10.72	.000	0.92	1.32	.78
Item 5 (Not many people seem to fully understand the importance of France)	1.12	0.11	10.47	.000	0.93	1.35	.80
Perceived similarity							
Item 1 (Similarity with Putin)	1.00				1.00	1.00	.94
Item 2 (Similarity with Russians)	0.74	0.08	9.64	.000	0.60	0.90	.63

Table S5

Regressions Analyses

Variable	*B*	*SE(B)*	*z*	*p*	95%CI		β
Judgment of Putin—Ukraine condition (R^2 = .26)							
National narcissism	-0.05	0.06	-0.80	.425	-0.17	0.07	-.05
Perceived similarity	0.37	0.07	5.19	.000	0.23	0.51	.54
Judgment of Putin—Ingroup condition (R^2 = .22)							
National narcissism	-0.08	0.07	-1.25	.213	-0.21	0.05	-.08
Perceived similarity	0.35	0.07	4.98	.000	0.21	0.49	.50
Perceived similarity (R^2 = .23)							
National narcissism	0.66	0.10	6.30	.000	0.45	0.86	.48

Table S6

Indirect and Total Effects of the Mediation Analysis on Judgement of Putin in the French Sample

Effect	*B*	*SE(B)*	*z*	*p*	95%CI		β
Indirect effects							
National narcissism → Similarity → Judgment of Putin—Ukraine condition (Indirect NN U)	0.24	0.06	4.19	.000	0.14	0.36	.25
National narcissism → Similarity → Judgment of Putin—Ingroup condition (Indirect NN I)	0.23	0.06	4.07	.000	0.13	0.35	.24
Contrast 1: Indirect NN U - Indirect NN I	0.01	0.02	0.81	.419	-0.02	0.05	.02
Total effects							
National narcissism → Judgment of Putin—Ukraine condition	0.19	0.07	2.75	.006	0.06	0.34	.20
National narcissism → Judgment of Putin—Ingroup condition	0.15	0.07	2.11	.035	0.02	0.29	.15

On Judgment of Russians

Figure S3

Tested Structural Equation Model on Judgment of Russians

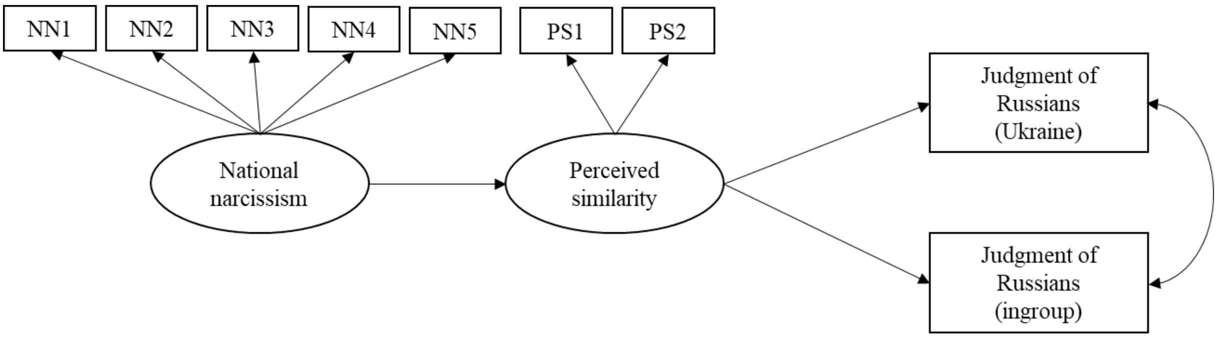

Note. Model fit: $\chi^2(23) = 81.43$, $p < .001$, CFI = .958, RMSEA = 0.089, 90%CI [0.069, 0.111], SRMR = 0.046

Table S7

Loadings of Items on Latent Variables

Variable/Item	*B*	*SE(B)*	*z*	*p*	95%CI		β
National narcissism							
Item 1 (France deserves special treatment)	1.00				1.00	1.00	.72
Item 2 (I will never be satisfied until France gets the recognition it deserves)	1.26	0.09	14.51	.000	1.12	1.46	.84
Item 3 (It makes me angry when people criticize France)	1.14	0.12	9.79	.000	0.93	1.39	.73
Item 4 (If France had a major say in the world, the world would be a much better place)	1.08	0.10	10.64	.000	0.90	1.30	.77
Item 5 (Not many people seem to fully understand the importance of France)	1.11	0.11	10.43	.000	0.92	1.34	.80
Perceived similarity							
Item 1 (Similarity with Putin)	1.00				1.00	1.00	.81
Item 2 (Similarity with Russians)	0.98	0.19	5.16	.000	0.67	1.42	.72

Table S8

Regressions Analyses

Variable	*B*	*SE(B)*	*z*	*p*	95%CI		β
Judgment of Russians—Ukraine condition (R^2 = .12)							
National narcissism	-6.46	1.91	-3.37	.001	-10.25	-2.69	-.28
Perceived similarity	7.15	2.42	2.95	.003	2.40	12.14	.37
Judgment of Russians—Ingroup condition (R^2 = .09)							
National narcissism	-7.43	1.97	-3.78	.000	-11.39	-3.65	-.30
Perceived similarity	5.55	2.24	2.48	.013	1.40	10.20	.27
Perceived similarity (R^2 = .24)							
National narcissism	0.58	0.13	4.40	.000	0.29	0.82	.48

Table S9

Indirect and Total Effects of Mediation on Judgement of Russians in the French Sample

Effect	*B*	*SE(B)*	*z*	*p*	95%CI		β
Indirect effects							
National narcissism → Similarity → Judgment of Russians—Ukraine condition (Indirect NN U)	4.16	1.36	3.05	.002	1.55	6.98	.18
National narcissism → Similarity → Judgment of Russians—Ingroup condition (Indirect NN I)	3.23	1.24	2.61	.009	0.88	5.73	.13
Contrast 1: Indirect NN U - Indirect NN I	0.93	0.71	1.32	.187	-0.34	2.45	.05
Total effects							
National narcissism → Judgment of Russians—Ukraine condition	-2.29	1.49	-1.54	.124	-5.46	0.41	-.10
National narcissism → Judgment of Russians—Ingroup condition	-4.20	1.63	-2.58	.010	-7.54	-1.17	-.17

Analyses on Social Judgment of Assertiveness (Study 1)

We conducted a mixed ANCOVA on the dimension of assertiveness of the social judgment of Putin, with the target (ingroup vs. Ukraine) as a within-subjects variable and national narcissism as predictor, see Table S10.

The target had a main effect, so that Putin was judged as more assertive in the Ukraine (*M* = 6.04, *SD* = 0.06) than in the ingroup condition (*M* = 5.93, *SD* = 0.07). National narcissism or its interaction with the target had no effect on the judgment of assertiveness, see also Table S8.

Table S10

Results of the ANCOVA on the Judgment of Assertiveness in the French Sample

Variable	$F(1,336)$	p	η^2_p
Target	5.32	.022	.02
Target x National narcissism	1.47	.226	.004
National narcissism	1.16	.282	.003

ANCOVA Controlling for National Satisfaction (Study 1)

On the Moral Judgment of the Attack

We conducted a mixed ANCOVA on the judgment of the Russian attack, with the target (ingroup vs. Ukraine; within-subjects), and *z*-scores of national narcissism and national satisfaction as predictors, see Tables S11-S12.

There was a main effect of the target. Although the attack was judged as immoral in both conditions, the judgment of immorality was less severe in the Ukraine condition (estimated *M* = 1.83, *SD* = 0.07) than the ingroup condition (estimated *M* = 1.59, *SD* = 0.06). As expected, the interaction national narcissism × target was significant. Specifically, national narcissism was more positively related to the moral judgment of the attack in the Ukraine than the ingroup condition. While there was no difference in judgment among low national

narcissists (at -1 *SD*; Ukraine target: *M* = 1.34, *SD* = 0.11; ingroup target: *M* = 1.39, *SD* = 0.09), *p* = .535, the attack on Ukraine was judged as more moral than on the ingroup among higher national narcissists (at +1 *SD*; Ukraine target: *M* = 2.32, *SD* = 0.11; ingroup target: *M* = 1.79, *SD* = 0.09), *p* < .001.

We also observed a negative main effect of national satisfaction and its interaction with the target was not significant.

Table S11

Results of the ANCOVA on Judgment of the Attack in the French Sample

Variable	*F*(1,336)	*p*	η^2_p
Target	23.75	<.001	.07
Target x National narcissism	23.72	<.001	.07
Target x National satisfaction	1.16	.283	.003
National narcissism	24.67	<.001	.07
National satisfaction	22.87	<.001	.06

Table S12

Effects of National Narcissism and National Satisfaction on Judgment of the Attack in the French Sample

Variable	*B*	*SE(B)*	*t*	*p*	95%CI		η^2_p
Judgment of the attack—Ukraine condition							
National narcissism	0.49	0.08	5.83	<.001	0.32	0.65	.09
National satisfaction	-0.36	0.08	-4.34	<.001	-0.53	-0.20	.05
Judgment of the attack—Ingroup condition							
National narcissism	0.20	0.07	3.05	.002	0.07	0.33	.03
National satisfaction	-0.30	0.07	-4.55	<.001	-0.43	-0.17	.06

On Social Judgment of Putin

We conducted the same model on the judgment of Putin, see Tables S13-S14. The target had a main effect, so that Putin was judged more positively in the Ukraine (estimated *M* = 2.54, *SD* = 0.07) than in the ingroup condition (estimated *M* = 2.34, *SD* = 0.07). National narcissism had a positive main effect whereas national satisfaction had a negative main effect

on judgment of Putin. The interactions of the target with national narcissism and with national satisfaction were not significant.

Table S13

Results of the ANCOVA on Judgment of Putin in The French Sample

Variable	*F*(1,336)	*p*	η^2_p
Target	63.07	<.001	.16
Target x National narcissism	0.67	.413	.002
Target x National satisfaction	0.31	.579	.001
National narcissism	12.72	<.001	.04
National satisfaction	8.47	.004	.03

Table S14

Effects of National Narcissism and National Satisfaction on Judgment of Putin in the French Sample

Variable	*B*	*SE(B)*	*t*	*p*	95%CI		η^2_p
Judgment of Putin—Ukraine condition							
National narcissism	0.30	0.08	3.62	<.001	0.14	0.46	.04
National satisfaction	-0.22	0.08	-2.72	.007	-0.38	-0.06	.02
Judgment of Putin—Ingroup condition							
National narcissism	0.27	0.08	3.39	.001	0.11	0.43	.03
National satisfaction	-0.24	0.08	-3.00	.003	-0.40	-0.08	.03

On Judgment of Russians

The same model was conducted on the judgment of Russians, see Tables S15-S16. Again, we found a main effect of the target with Russians being judged more positively when the target was Ukraine (estimated *M* = 42.87, *SD* = 1.61) than when it was the ingroup (estimated *M* = 32.88, *SD* = 1.72). Moreover, the interaction of the target with national satisfaction was significant. Specifically, national satisfaction was related to a more positive judgment of Russians when the target was Ukraine but not when it was the ingroup. At lower levels of satisfaction (-1 *SD*), Russians were judged more positively in the Ukraine condition (*M* = 38.69, *SD* = 2.53) than in the ingroup condition (*M* = 32.18, *SD* = 2.71), $p < .001$. The

same pattern was observed at elevated levels of satisfaction (*+1 SD*; Ukraine target: M = 47.04, SD = 2.53; ingroup target: M = 33.57, SD = 2.70). The analysis revealed no significant effects of the interaction of the target with national narcissism. However, national narcissism had a negative main effect on the judgement of Russians.

Table S15

Results of the ANCOVA on Judgment of Russians in the French Sample

Variable	$F(1,336)$	p	η^2_p
Target	90.17	<.001	.22
Target x National narcissism	0.03	.856	.00
Target x National satisfaction	7.50	.007	.02
National narcissism	8.08	.005	.02
National satisfaction	1.62	.204	.01

Table S16

Effects of National Narcissism and National Satisfaction on Judgment of Russians in the French Sample

Variable	B	$SE(B)$	t	p	95%CI		η^2_p
Judgment of Russians—Ukraine condition							
National narcissism	-5.40	1.98	-2.73	.007	-9.29	-1.51	.02
National satisfaction	4.18	1.95	2.14	.033	0.34	8.01	.01
Judgment of Russians—Ingroup condition							
National narcissism	-5.64	2.11	-2.67	.008	-9.79	-1.48	.02
National satisfaction	0.70	2.08	0.34	.737	-3.39	4.79	.00

Structural Equation Model Analyses with National Satisfaction (Study 1)

On Moral Judgment of the Attack

Figure S4

Tested Structural Equation Model on Judgment of the Attack

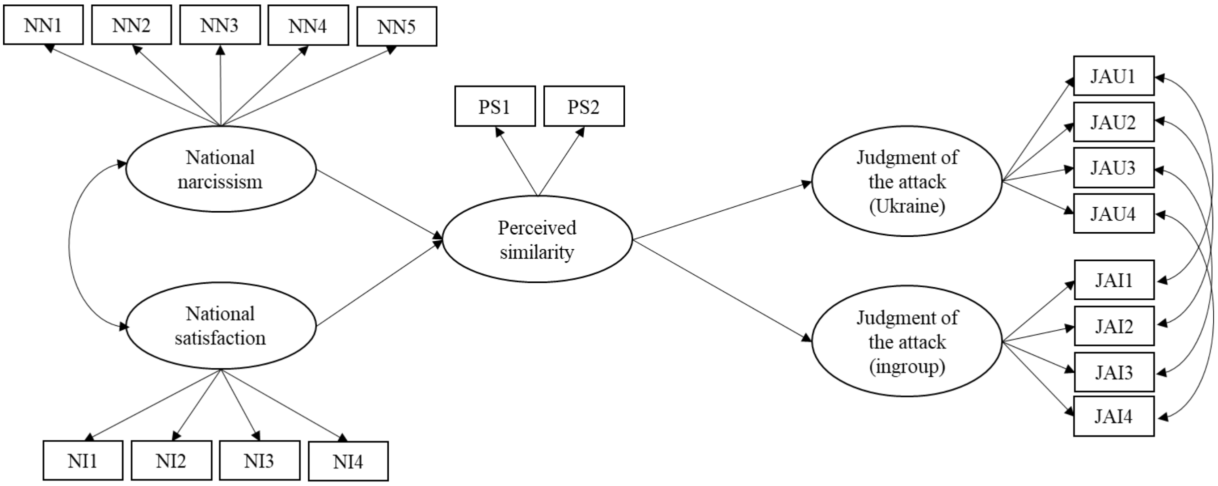

Note. Model fit: $X^2(138) = 267.92$, $p < .001$, CFI = .970, RMSEA = 0.053, 90%CI [0.044, 0.063], SRMR = 0.047

Table S17

Loadings of Items on Latent Variables

Variable/Item	*B*	*SE(B)*	*z*	*p*	95%CI		β
Judgment of the attack—Ukraine condition							
Item 1 (justified)	1.00				1.00	1.00	.89
Item 2 (moral)	0.67	0.06	12.08	<.001	0.56	0.78	.78
Item 3 (legitimate)	1.00	0.04	26.37	<.001	0.93	1.08	.95
Item 4 (severe)	-0.62	0.06	-10.20	<.001	-0.74	-0.50	-.66
Judgment of the attack—Ingroup condition							
Item 1 (justified)	1.00				1.00	1.00	.87
Item 2 (moral)	0.61	0.07	8.69	<.001	0.46	0.74	.77
Item 3 (legitimate)	0.97	0.05	19.95	<.001	0.89	1.08	.96
Item 4 (severe)	-0.46	0.09	-5.13	<.001	-0.65	-0.30	-.48
National narcissism							
Item 1 (France deserves special treatment)	1.00				1.00	1.00	.73
Item 2 (I will never be satisfied until France gets the recognition it deserves)	1.24	0.08	14.63	<.001	1.10	1.43	.84
Item 3 (It makes me angry when people criticize France)	1.14	0.11	10.08	<.001	0.95	1.39	.74
Item 4 (If France had a major say in the world, the world would be a much better place)	1.10	0.10	11.47	<.001	0.94	1.31	.79
Item 5 (Not many people seem to fully understand the importance of France)	1.12	0.10	11.51	<.001	0.95	1.33	.80

National satisfaction							
Item 1 (I am glad to be French)	1.00				1.00	1.00	.89
Item 2 (I think that French people have a lot to be proud of)	1.08	0.05	21.86	<.001	0.99	1.18	.85
Item 3 (It is pleasant to be French)	1.03	0.04	24.94	<.001	0.96	1.12	.89
Item 4 (Being French gives me a good feeling)	1.07	0.06	19.33	<.001	0.97	1.19	.84
Perceived similarity							
Item 1 (Similarity with Putin)	1.00				1.00	1.00	.93
Item 2 (Similarity with Russians)	0.75	0.09	8.26	<.001	0.58	0.94	.63

Table S18

Regressions Analyses of Model

Variable	*B*	*SE(B)*	*z*	*p*	95%CI		β
Judgment of the attack—Ukraine condition (R^2 = .34)							
National narcissism	0.29	0.09	3.23	.001	0.13	0.48	.24
National satisfaction	-0.41	0.08	-5.13	<.001	-0.57	-0.25	-.38
Perceived similarity	0.45	0.08	5.41	<.001	0.30	0.63	.50
Judgment of the attack—Ingroup condition (R^2 = .18)							
National narcissism	0.10	0.07	1.55	.122	-0.02	0.24	.10
National satisfaction	-0.31	0.08	-4.13	<.001	-0.47	-0.17	-.35
Perceived similarity	0.28	0.06	4.86	<.001	0.17	0.40	.38
Perceived similarity (R^2 = .23)							
National narcissism	0.57	0.12	4.59	<.001	0.33	0.82	.42
National satisfaction	0.12	0.10	1.25	.213	-0.07	0.31	.10

Table S19

Indirect and Total Effects of the Mediation Analysis on Judgement of the Attack in French Sample

Effect	*B*	*SE(B)*	*z*	*p*	95%CI		β
Indirect effects							
National narcissism → Similarity → Judgment of the attack—Ukraine condition (Indirect NN U)	0.26	0.07	3.80	<.001	0.14	0.40	.21
National narcissism → Similarity → Judgment of the attack—Ingroup condition (Indirect NN I)	0.16	0.05	3.54	<.001	0.08	0.26	.16
Contrast 1: Indirect NN U - Indirect NN I	0.10	0.04	2.67	.008	0.04	0.18	.05
National satisfaction → Similarity → Judgment of the attack—Ukraine condition (Indirect NS U)	0.05	0.05	1.15	.250	-0.03	0.16	.05
National satisfaction → Similarity → Judgment of the attack—Ingroup condition (Indirect NS I)	0.03	0.03	1.13	.261	-0.02	0.10	.04
Contrast 2: Indirect NS U - Indirect NS I	0.02	0.02	1.09	.277	-0.01	0.06	.01
Total effects							
National narcissism → Judgment of the attack—Ukraine condition	0.55	0.11	5.18	<.001	0.35	0.78	.45
National narcissism → Judgment of the attack—Ingroup condition	0.26	0.07	3.52	<.001	0.12	0.41	.26
National satisfaction → Judgment of the attack—Ukraine condition	-0.36	0.09	-4.12	<.001	-0.53	-0.18	-.33
National satisfaction → Judgment of the attack—Ingroup condition	-0.28	0.08	-3.65	<.001	-0.44	-0.13	-.32

On Social Judgment of Putin

Figure S5

Tested Structural Equation Model on Judgment of Putin

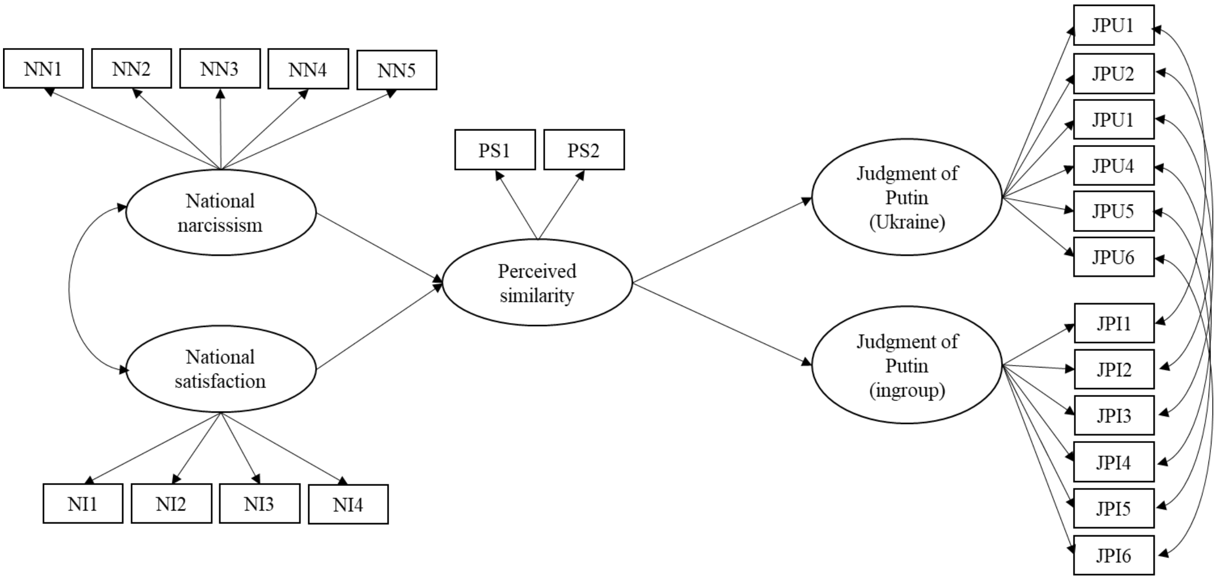

Note. Model fit: $\chi^2(214) = 589.55$, $p < .001$, CFI = .945, RMSEA = 0.073, 90%CI [0.066, 0.080], SRMR = 0.064

Table S20

Loadings of Items on Latent Variables

Variable/Item	*B*	*SE(B)*	*z*	*p*	95%CI		β
Judgment of Putin—Ukraine condition							
Item 1 (competent)	1.00				1.00	1.00	.62
Item 2 (intelligent)	0.71	0.06	12.06	<.001	0.60	0.83	.45
Item 3 (friendly)	0.99	0.08	12.77	<.001	0.85	1.16	.89
Item 4 (empathetic)	0.87	0.09	10.20	<.001	0.72	1.05	.79
Item 5 (fair)	1.04	0.08	12.46	<.001	0.89	1.22	.89
Item 6 (trustworthy)	1.14	0.09	13.16	<.001	0.98	1.32	.87
Judgment of Putin—Ingroup condition							
Item 1 (competent)	1.00				1.00	1.00	.60
Item 2 (intelligent)	0.77	0.05	15.92	<.001	0.67	0.86	.45
Item 3 (friendly)	0.96	0.08	12.70	<.001	0.82	1.13	.92
Item 4 (empathetic)	0.82	0.07	11.32	<.001	0.67	0.96	.80
Item 5 (fair)	0.95	0.09	10.67	<.001	0.79	1.14	.86
Item 6 (trustworthy)	1.13	0.08	13.42	<.001	0.98	1.31	.91

National narcissism							
Item 1 (France deserves special treatment)	1.00				1.00	1.00	.72
Item 2 (I will never be satisfied until France gets the recognition it deserves)	1.25	0.09	14.38	<.001	1.11	1.45	.83
Item 3 (It makes me angry when people criticize France)	1.16	0.12	9.76	<.001	0.96	1.42	.74
Item 4 (If France had a major say in the world, the world would be a much better place)	1.12	0.10	11.05	<.001	0.95	1.34	.79
Item 5 (Not many people seem to fully understand the importance of France)	1.15	0.11	10.86	<.001	0.96	1.37	.81
National satisfaction							
Item 1 (I am glad to be French)	1.00				1.00	1.00	.89
Item 2 (I think that French people have a lot to be proud of)	1.09	0.05	21.60	<.001	0.99	1.19	.85
Item 3 (It is pleasant to be French)	1.04	0.04	24.96	<.001	0.96	1.12	.89
Item 4 (Being French gives me a good feeling)	1.09	0.06	19.29	<.001	0.98	1.20	.84
Perceived similarity							
Item 1 (Similarity with Putin)	1.00				1.00	1.00	.90
Item 2 (Similarity with Russians)	0.81	0.08	10.29	<.001	0.66	0.97	.66

Table S21

Regressions Analyses of Model

Variable	*B*	*SE(B)*	*z*	*p*	95%CI		β
Judgment of Putin—Ukraine condition (R^2 = .35)							
National narcissism	0.09	0.07	1.34	.182	-0.04	0.24	.10
National satisfaction	-0.28	0.07	-4.28	<.001	-0.42	-0.16	-.33
Perceived similarity	0.43	0.07	5.88	<.001	0.29	0.58	.60
Judgment of Putin—Ingroup condition (R^2 = .33)							
National narcissism	0.08	0.07	1.20	.231	-0.05	0.22	.09
National satisfaction	-0.31	0.07	-4.69	<.001	-0.45	-0.18	-.37
Perceived similarity	0.41	0.07	5.60	<.001	0.27	0.56	.56
Perceived similarity (R^2 = .25)							
National narcissism	0.57	0.13	4.37	<.001	0.32	0.84	.43
National satisfaction	0.13	0.10	1.33	.182	-0.06	0.32	.11

Table S22

Indirect and Total Effects of the Mediation Analysis on Judgement of Putin in the French Sample

Effect	*B*	*SE(B)*	*z*	*p*	95%CI		β
Indirect effects							
National narcissism → Similarity → Judgment of Putin—Ukraine condition (Indirect NN U)	0.25	0.06	3.94	<.001	0.13	0.38	.26
National narcissism → Similarity → Judgment of Putin—Ingroup condition (Indirect NN I)	0.23	0.06	3.88	<.001	0.12	0.36	.24
Contrast 1: Indirect NN U - Indirect NN I	*0.01*	*0.02*	*0.74*	*.457*	*-0.02*	*0.05*	*.01*
National satisfaction → Similarity → Judgment of Putin—Ukraine condition (Indirect NS U)	0.06	0.05	1.24	.216	-0.03	0.15	.07
National satisfaction → Similarity → Judgment of Putin—Ingroup condition (Indirect NS I)	0.05	0.04	1.23	.219	-0.02	0.14	.06
Contrast 2: Indirect NS U - Indirect NS I	*0.00*	*0.01*	*0.56*	*.575*	*-0.01*	*0.02*	*.00*
Total effects							
National narcissism → Judgment of Putin—Ukraine condition	0.34	0.09	3.93	<.001	0.18	0.52	.35
National narcissism → Judgment of Putin—Ingroup condition	0.32	0.08	3.80	<.001	0.16	0.48	.33
National satisfaction → Judgment of Putin—Ukraine condition	-0.23	0.07	-3.17	.002	-0.37	-0.09	-.27
National satisfaction → Judgment of Putin—Ingroup condition	-0.26	0.07	-3.69	<.001	-0.40	-0.12	-.31

On Judgment of Russians

Figure S6

Tested Structural Equation Model on Judgment of Russians

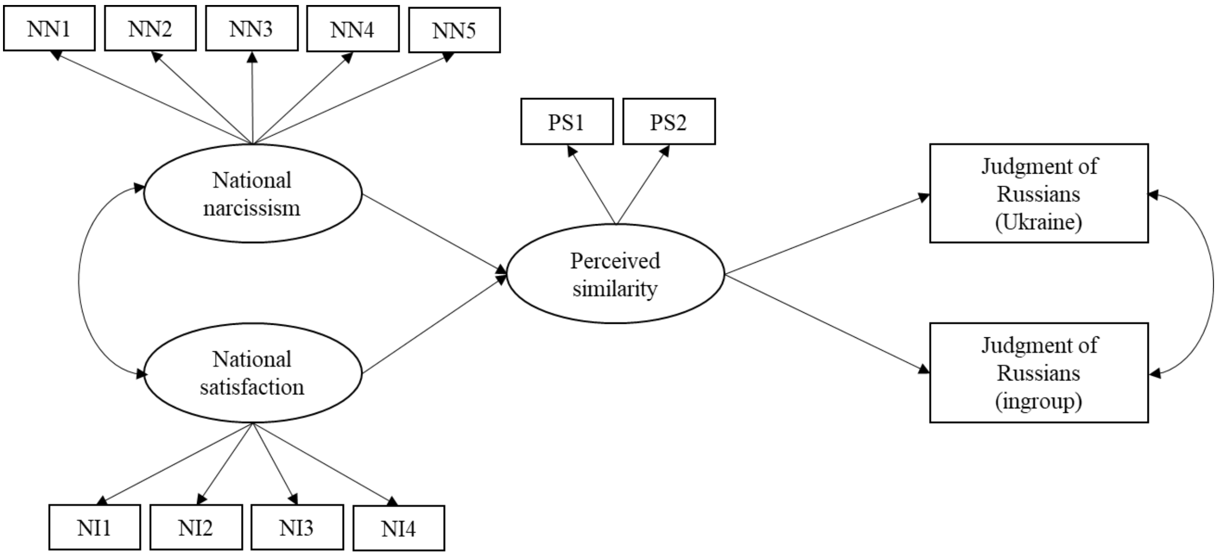

Note. Model fit: $\chi^2(57) = 175.37$, $p < .001$, CFI = .95, RMSEA = 0.081, 90%CI [0.067, 0.096], SRMR = 0.053

Table S23

Loadings of Items on Latent Variables

Variable/Item	*B*	*SE(B)*	*z*	*p*	95%CI		β
National narcissism							
Item 1 (France deserves special treatment)	1.00				1.00	1.00	0.71
Item 2 (I will never be satisfied until France gets the recognition it deserves)	1.26	0.09	14.14	<.001	1.12	1.47	0.83
Item 3 (It makes me angry when people criticize France)	1.17	0.12	9.82	<.001	0.96	1.44	0.75
Item 4 (If France had a major say in the world, the world would be a much better place)	1.11	0.10	10.96	<.001	0.94	1.33	0.78
Item 5 (Not many people seem to fully understand the importance of France)	1.14	0.10	10.96	<.001	0.96	1.37	0.80

National satisfaction							
Item 1 (I am glad to be French)	1.00				1.00	1.00	0.89
Item 2 (I think that French people have a lot to be proud of)	1.07	0.05	20.62	<.001	0.98	1.18	0.85
Item 3 (It is pleasant to be French)	1.03	0.04	24.76	<.001	0.95	1.12	0.89
Item 4 (Being French gives me a good feeling)	1.09	0.06	18.33	<.001	0.98	1.21	0.84
Perceived similarity							
Item 1 (Similarity with Putin)	1.00				1.00	1.00	0.80
Item 2 (Similarity with Russians)	1.00	0.20	5.04	<.001	0.70	1.45	0.73

Table S24

Regressions Analyses

Variable	*B*	*SE(B)*	*z*	*p*	95%CI		β
Judgment of Russians—Ukraine condition (R^2 = .13)							
National narcissism	-7.98	2.27	-3.52	.000	-12.67	-3.71	-.34
National satisfaction	2.10	1.68	1.25	.212	-1.18	5.40	.10
Perceived similarity	7.19	2.68	2.68	.007	2.63	11.89	.37
Judgment of Russians—Ingroup condition (R^2 = .09)							
National narcissism	-7.55	2.28	-3.32	.001	-12.08	-3.29	-.30
National satisfaction	-0.30	1.70	-0.18	.858	-3.62	2.96	-.01
Perceived similarity	5.85	2.62	2.23	.026	1.55	10.51	.28
Perceived similarity (R^2 = .25)							
National narcissism	0.48	0.16	2.92	.003	0.16	0.80	.40
National satisfaction	0.16	0.10	1.64	.101	-0.04	0.34	.15

Table S25

Indirect and Total Effects of Mediation on Judgement of Russians in the French Sample

Effect	*B*	*SE(B)*	*z*	*p*	95%CI		β
Indirect effects							
National narcissism → Similarity → Judgment of Russians—Ukraine condition (Indirect NN U)	3.45	1.34	2.57	.010	1.08	6.32	.15
National narcissism → Similarity → Judgment of Russians—Ingroup condition (Indirect NN I)	2.81	1.20	2.34	.019	0.66	5.28	.11
Contrast 1: Indirect NN U - Indirect NN I	0.64	0.63	1.02	.308	-0.39	2.13	.04
National satisfaction → Similarity → Judgment of Russians—Ukraine condition (Indirect NS U)	1.12	0.85	1.32	.186	-0.23	2.87	.06
National satisfaction → Similarity → Judgment of Russians—Ingroup condition (Indirect NS I)	0.91	0.75	1.23	.221	-0.17	2.50	.04
Contrast 2: Indirect NS U - Indirect NS I	0.21	0.25	0.83	.407	-0.17	0.81	.01
Total effects							
National narcissism → Judgment of Russians—Ukraine condition	-4.54	2.06	-2.20	.028	-8.81	-0.72	-.19
National narcissism → Judgment of Russians—Ingroup condition	-4.74	2.12	-2.23	.026	-9.12	-0.85	-.19
National satisfaction → Judgment of Russians—Ukraine condition	3.22	1.66	1.95	.052	-0.02	6.44	.16
National satisfaction → Judgment of Russians—Ingroup condition	0.61	1.69	0.36	.718	-2.74	3.99	.03

Procedure Details (Study 2)

Ukraine Target Condition

Participants were reminded of Russia's military action in Ukraine via this paragraph from Agence France Presse, "Shortly before 3am GMT on the night of Wednesday 23 February 2022, Russian President Vladimir Putin appeared on Russian television and announced that he had 'taken the decision on a military operation' in Ukraine, officially to defend Russian separatists. In the aftermath, bombing raids hit the whole of Ukraine, including the capital Kiev. Russian ground troops also entered the country."

Ingroup (US) Target Condition

Participants were asked to imagine a similar attack on American soil using the following paragraph, "Now we would like you to take a few seconds to imagine an attack like the one in Ukraine happening in your country, the United States. During the night, the Russian president, Vladimir Putin announces an attack on your country. Russian planes bomb major cities in the US and the Russian army arrives on American soil."

Complete Results of the Structural Equation Model Analyses (Study 2)

On Moral Judgment of the Attack

Table S26

Loadings of Items on Latent Variables in the American Sample

Variable/Item	*B*	*SE(B)*	*z*	*p*	95%CI		β
Judgment of the attack—Ukraine condition							
Item 1 (justified)	1.00				1.00	1.00	.93
Item 2 (moral)	0.71	0.05	13.56	.000	0.60	0.81	.90
Item 3 (legitimate)	0.90	0.05	16.61	.000	0.79	1.01	.81
Item 4 (severe)	-0.64	0.09	-7.00	.000	-0.84	-0.48	-.61
Judgment of the attack—Ingroup condition							
Item 1 (justified)	1.00				1.00	1.00	.81
Item 2 (moral)	0.80	0.06	12.37	.000	0.67	0.93	.92
Item 3 (legitimate)	0.86	0.06	15.47	.000	0.74	0.96	.79
Item 4 (severe)	-0.42	0.10	-4.27	.000	-0.63	-0.25	-.30
National narcissism							
Item 1 (the United States deserves special treatment)	1.00				1.00	1.00	.79
Item 2 (I will never be satisfied until the United States gets the recognition it deserves)	0.94	0.04	22.29	.000	0.85	1.02	.83
Item 3 (It makes me angry when people criticize the United States)	1.10	0.08	13.41	.000	0.95	1.27	.78
Item 4 (If the United States had a major say in the world, the world would be a much better place)	1.06	0.07	15.67	.000	0.94	1.20	.79
Item 5 (Not many people seem to fully understand the importance of the United States)	1.09	0.08	13.27	.000	0.94	1.26	.77
Perceived similarity							
Item 1 (Similarity with Putin)	1.00				1.00	1.00	1.10
Item 2 (Similarity with Russians)	0.43	0.10	4.35	.000	0.22	0.61	.47

Table S27

Regressions Analyses

Variable	*B*	*SE(B)*	*z*	*p*	95%CI		β
Judgment of the attack—Ukraine condition (R^2 = .09)							
National narcissism	0.08	0.05	1.43	.154	-0.03	0.18	.10
Perceived similarity	0.12	0.05	2.60	.009	0.04	0.22	.25
Judgment of the attack—Ingroup condition (R^2 = .04)							
National narcissism	0.09	0.05	1.62	.104	-0.02	0.19	.12
Perceived similarity	0.06	0.04	1.54	.123	0.00	0.14	.13
Perceived similarity (R^2 = .16)							
National narcissism	0.63	0.08	7.51	.000	0.47	0.80	.39

Table S28

Indirect and Total Effects for the Mediation on Judgement of the Attack in the American Sample

Effect	*B*	*SE(B)*	*z*	*p*	95%CI		β
Indirect effects							
National narcissism → Similarity → Judgment of the attack—Ukraine condition (Indirect NN U)	0.08	0.03	2.35	.019	0.02	0.15	.10
National narcissism → Similarity → Judgment of the attack—Ingroup condition (Indirect NN I)	0.04	0.02	1.46	.143	0.00	0.09	.05
Contrast 1: Indirect NN U - Indirect NN I	0.04	0.02	2.16	.031	0.01	0.08	.05
Total effects							
National narcissism → Judgment of the attack—Ukraine condition	0.15	0.05	3.03	.002	0.06	0.25	.20
National narcissism → Judgment of the attack—Ingroup condition	0.12	0.05	2.53	.012	0.03	0.22	.17

On Social Judgment of Putin

Table S29

Loadings of Items on Latent Variables in the American Sample

Variable/Item	*B*	*SE(B)*	*z*	*p*	95%CI		β
Judgment of Putin—Ukraine condition							
Item 1 (competent)	1.00				1.00	1.00	.48
Item 2 (intelligent)	0.82	0.06	13.74	.000	0.71	0.94	.40
Item 3 (friendly)	0.95	0.10	9.48	.000	0.79	1.18	.79
Item 4 (empathetic)	0.91	0.11	8.15	.000	0.72	1.16	.84
Item 5 (fair)	0.88	0.12	7.43	.000	0.67	1.13	.86
Item 6 (trustworthy)	0.80	0.13	6.16	.000	0.57	1.08	.82
Judgment of Putin—Ingroup condition							
Item 1 (competent)	1.00				1.00	1.00	.34
Item 2 (intelligent)	1.00	0.10	10.45	.000	0.84	1.21	.32
Item 3 (friendly)	0.98	0.18	5.51	.000	0.75	1.44	.82
Item 4 (empathetic)	0.86	0.20	4.33	.000	0.60	1.35	.83
Item 5 (fair)	0.97	0.23	4.31	.000	0.67	1.53	.78
Item 6 (trustworthy)	0.77	0.18	4.18	.000	0.51	1.21	.79
National narcissism							
Item 1 (the United States deserves special treatment)	1.00				1.00	1.00	.80
Item 2 (I will never be satisfied until the United States gets the recognition it deserves)	0.94	0.04	22.33	.000	0.85	1.01	.83
Item 3 (It makes me angry when people criticize the United States)	1.10	0.08	13.93	.000	0.96	1.27	.78
Item 4 (If the United States had a major say in the world, the world would be a much better place)	1.05	0.07	15.93	.000	0.93	1.19	.79
Item 5 (Not many people seem to fully understand the importance of the United States)	1.09	0.08	13.35	.000	0.94	1.27	.77
Perceived similarity							
Item 1 (Similarity with Putin)	1.00				1.00	1.00	1.00
Item 2 (Similarity with Russians)	0.53	0.09	6.15	.000	0.35	0.69	.51

Table S30

Regressions Analyses

Variable	*B*	*SE(B)*	*z*	*p*	95%CI		β
Judgment of Putin—Ukraine condition (R^2 = .16)							
National narcissism	0.07	0.05	1.50	.135	-0.02	0.17	.10
Perceived similarity	0.17	0.05	3.68	.000	0.08	0.27	.35
Judgment of Putin—Ingroup condition (R^2 = .15)							
National narcissism	0.08	0.04	1.99	.046	0.00	0.15	.14
Perceived similarity	0.11	0.04	2.59	.010	0.04	0.20	.30
Perceived similarity (R^2 = .19)							
National narcissism	0.64	0.08	7.66	.000	0.48	0.80	.44

Table S31

Indirect and Total Effects for the Mediation on Judgment of Putin in the American Sample

Effect	*B*	*SE(B)*	*z*	*p*	95%CI		β
Indirect effects							
National narcissism → Similarity → Judgment of Putin—Ukraine condition (Indirect NN U)	0.11	0.03	3.33	.001	0.05	0.18	.15
National narcissism → Similarity → Judgment of Putin—Ingroup condition (Indirect NN I)	0.07	0.03	2.46	.014	0.02	0.13	.13
Contrast 1: Indirect NN U - Indirect NN I	0.04	0.01	2.80	.005	0.01	0.07	.02
Total effects							
National narcissism → Judgment of Putin—Ukraine condition	0.18	0.05	3.71	.000	0.09	0.28	.25
National narcissism → Judgment of Putin—Ingroup condition	0.15	0.04	3.55	.000	0.07	0.23	.27

On Judgment of Russians

Judgment of Russians.

The model had an adequate fit, $X^2(57) = 203.46$, $p < .001$, CFI = .959, RMSEA = 0.081, 90%CI [0.069, 0.093], SRMR = 0.042.

Table S32

Loadings of Items on Latent Variables in the American Sample

Variable/Item	*B*	*SE(B)*	*z*	*p*	95%CI		β
National narcissism							
Item 1 (the United States deserves special treatment)	1.00				1.00	1.00	.79
Item 2 (I will never be satisfied until the United States gets the recognition it deserves)	0.94	0.04	22.48	.000	0.85	1.02	.82
Item 3 (It makes me angry when people criticize the United States)	1.11	0.08	13.19	.000	0.96	1.29	.78
Item 4 (If the United States had a major say in the world, the world would be a much better place)	1.06	0.07	15.07	.000	0.94	1.21	.79
Item 5 (Not many people seem to fully understand the importance of the United States)	1.09	0.08	12.92	.000	0.94	1.26	.77
Perceived similarity							
Item 1 (Similarity with Putin)	1.00				1.00	1.00	1.00
Item 2 (Similarity with Russians)	0.52	0.12	4.48	.000	0.30	0.75	.51

Table S33

Regressions Analyses

Variable	*B*	*SE(B)*	*z*	*p*	95%CI		β
Judgment of Russians—Ukraine condition (R^2 = .04)							
National narcissism	-1.75	1.33	-1.31	.189	-4.48	0.78	-.09
Perceived similarity	2.11	1.14	1.86	.064	0.35	4.78	.15
Judgment of Russians —Ingroup condition (R^2 = .03)							
National narcissism	-3.02	1.42	-2.13	.033	-5.85	-0.23	-.13
Perceived similarity	1.92	1.11	1.73	.084	0.13	4.43	.13
Perceived similarity (R^2 = .28)							
National narcissism	0.64	0.09	7.34	.000	0.46	0.81	.43

Table S34

Indirect and Total Effects of the Mediation on Judgment of Russians in the American Sample

Effect	*B*	*SE(B)*	*z*	*p*	95%CI		β
Indirect effects							
National narcissism → Similarity → Judgment of Russians—Ukraine condition (Indirect NN U)	1.35	0.73	1.86	.063	0.22	3.01	.07
National narcissism → Similarity → Judgment of Russians—Ingroup condition (Indirect NN I)	1.23	0.73	1.68	.093	0.07	2.90	.06
Contrast 1: Indirect NN U - Indirect NN I	0.13	0.40	0.32	.753	-0.58	1.02	.01
Total effects							
National narcissism → Judgment of Russians—Ukraine condition	-0.40	1.10	-0.36	.716	-2.48	1.80	-.02
National narcissism → Judgment of Russians—Ingroup condition	-1.80	1.22	-1.47	.141	-4.06	0.68	-.08

Analyses on Social Judgment of Assertiveness (Study 2)

We conducted a mixed ANCOVA on the dimension of assertiveness of the social judgment of Putin, with the target (ingroup vs. Ukraine) as a within-subjects variable and collective narcissism and ingroup satisfaction as predictors, see Table S25. No significant effect was observed.

Table S35

Results of the ANCOVA on Judgment of Assertiveness in the American Sample

Variable	$F(1,394)$	p	η^2_p
Target	0.37	.365	.002
Target x National narcissism	0.67	.667	.00
National narcissism	0.19	.667	.00

ANCOVA Controlling for National Satisfaction (Study 2)

On Moral Judgment of the Attack

We conducted a mixed ANCOVA on the judgment of the Russian attack, with the target (ingroup vs. Ukraine; within-subjects), and *z*-scores of national narcissism and national satisfaction as predictors, see Tables SXX-SXX. The target had no main effect on the judgement of the attack. National narcissism was related to a less immoral judgment of the attack, regardless of the condition. However, the interaction between national satisfaction and the target was significant. More precisely, national satisfaction was not related to the moral judgment of the attack when the target was Ukraine but was negatively related to the moral judgment of the attack when the target was the US. At low levels of national satisfaction (-1 *SD*), participants judged the attack on Ukraine (M = 1.36, SD = 0.06) as more immoral than the attack on the ingroup (M = 1.51, SD = 0.06), p = .011. In contrast, higher identifiers (+ 1

SD) did not judge differently the attack on Ukraine (*M* = 1.38, *SD* = 0.06) compared to an attack on the ingroup (*M* = 1.29, *SD* = 0.06), *p* = .092.

Table S36

Results of the ANCOVA on Judgment of the Attack in the American Sample

Variable	*F*(1,394)	*p*	η^2_p
Target	0.51	.477	.001
Target x National narcissism	0.49	.486	.001
Target x National satisfaction	7.29	.007	.02
National narcissism	15.88	<.001	.04
National satisfaction	1.45	.230	.004

Table S37

Effects of National Narcissism and National Satisfaction on Judgment of the Attack in the American Sample

Variable	*B*	*SE(B)*	*t*	*p*	95%CI		η^2_p
Judgment of the attack—Ukraine condition							
National narcissism	0.15	0.05	3.05	.002	0.05	0.24	.02
National satisfaction	0.01	0.05	0.23	.816	-0.08	0.11	.00
Judgment of the attack—Ingroup condition							
National narcissism	0.18	0.05	3.99	<.001	0.09	0.27	.04
National satisfaction	-0.11	0.05	-2.45	.002	-0.20	-0.02	.02

On the Social Judgment of Putin

We conducted the same mixed ANCOVA on the social judgment of Putin. The target had a main effect, with Putin being judged more negatively when the target was the ingroup (estimated *M* = 2.27, *SD* = 0.04) than Ukraine (estimated *M* = 1.95, *SD* = 0.04). Contrary to our expectations, national narcissism was not associated with the judgment of Putin, and this was not moderated by the target. However, national satisfaction had a significant and positive main effect of the judgment of Putin that was not moderated by the target.

Table S38

Results of the ANCOVA on Judgment of Putin in the American Sample

Variable	F(1,394)	p	η^2_p
Target	125.35	<.001	.24
Target x National narcissism	2.82	.094	.01
Target x National satisfaction	0.83	.364	.002
National narcissism	1.54	.215	.004
National satisfaction	12.21	.001	.03

Table S39

Effects of National Narcissism and National Satisfaction on Judgment of Putin in the American Sample

Variable	B	$SE(B)$	t	p	95%CI		η^2_p
Judgment of Putin—Ukraine condition							
National narcissism	0.09	0.06	1.65	.099	-0.02	0.20	.01
National satisfaction	0.16	0.06	2.80	.005	0.05	0.26	.02
Judgment of Putin—Ingroup condition							
National narcissism	0.03	0.05	0.62	.536	-0.07	0.13	.001
National satisfaction	0.19	0.05	3.82	<.001	0.09	0.29	.04

On Judgment of Russians

Finally, we conducted the same analyses on the judgment of Russians. The target had a main effect, with a more positive judgment of Russians in the Ukraine target condition (estimated M = 44.22, SD = 1.91) than in the ingroup condition (estimated M = 31.15, SD = 1.32). National narcissism was negatively related to the judgment of Russians, whereas national satisfaction was positively related to the judgment of Russians. These main effects were not moderated by the target.

Table S40

Results of the ANCOVA on Judgment of Russians in the American Sample

Variable	F(1,394)	p	η^2_p
Target	225.81	<.001	.37
Target x National narcissism	1.15	.285	.00
Target x National satisfaction	0.46	.498	.00

National narcissism	4.32	.038	.01
National satisfaction	4.00	.046	.01

Table S41

Effects of National Narcissism and National Satisfaction on Judgment of Russians in the American Sample

Variable	*B*	*SE(B)*	*t*	*p*	95%CI		η^2_p
Judgment of Russians—Ukraine condition							
National narcissism	-2.54	1.53	-1.66	.097	-5.54	0.46	.01
National satisfaction	3.38	1.52	2.22	.027	0.39	6.37	.01
Judgment of Russians—Ingroup condition							
National narcissism	-3.73	1.69	-2.21	.027	-7.05	-0.42	.01
National satisfaction	2.63	1.68	1.57	.118	-0.67	5.93	.01

Structural Equation Model Analyses with National Satisfaction (Study 2)

On Moral Judgment of the Attack

The model conducted on the judgment of the attack had an adequate fit, $X^2(138) = 338.85$, $p < .001$, CFI = .963, RMSEA = 0.060, 90%CI [0.052, 0.068], SRMR = 0.049.

Table S42

Loadings of Items on Latent Variables in the American Sample

Variable/Item	*B*	*SE(B)*	*z*	*p*	95%CI		β
Judgment of the attack—Ukraine condition							
Item 1 (justified)	1.00				1.00	1.00	.93
Item 2 (moral)	0.71	0.05	13.42	<.001	0.61	0.81	.89
Item 3 (legitimate)	0.90	0.06	16.51	<.001	0.79	1.01	.81
Item 4 (severe)	-0.64	0.09	-7.02	<.001	-0.83	-0.48	-.61
Judgment of the attack—Ingroup condition							
Item 1 (justified)	1.00				1.00	1.00	.81
Item 2 (moral)	0.79	0.06	12.60	<.001	0.66	0.91	.92
Item 3 (legitimate)	0.86	0.06	15.58	<.001	0.74	0.96	.79
Item 4 (severe)	-0.41	0.10	-4.17	<.001	-0.64	-0.24	-.30
National narcissism							
Item 1 (the United States deserves special treatment)	1.00				1.00	1.00	.77
Item 2 (I will never be satisfied until	0.93	0.04	22.13	<.001	0.85	1.01	.80

the United States gets the recognition it deserves)							
Item 3 (It makes me angry when people criticize the United States)	1.17	0.09	13.73	<.001	1.02	1.35	.80
Item 4 (If the United States had a major say in the world, the world would be a much better place)	1.12	0.07	15.60	<.001	0.99	1.27	.81
Item 5 (Not many people seem to fully understand the importance of the United States)	1.14	0.08	13.73	<.001	0.99	1.32	.78
National satisfaction							
Item 1 (I am glad to be American)	1.00				1.00	1.00	.92
Item 2 (I think that Americans have a lot to be proud of)	0.97	0.03	36.17	<.001	0.92	1.03	.90
Item 3 (It is pleasant to be American)	0.97	0.03	30.55	<.001	0.91	1.03	.87
Item 4 (Being American gives me a good feeling)	1.07	0.03	35.77	<.001	1.02	1.14	.95
Perceived similarity							
Item 1 (Similarity with Putin)	1.00				1.00	1.00	.95
Item 2 (Similarity with Russians)	0.58	0.10	6.02	<.001	0.39	0.77	.54

Table S43

Regressions Analyses

Variable	*B*	*SE(B)*	*z*	*p*	95%CI		β
Judgment of the attack—Ukraine condition (R^2 = .12)							
National narcissism	0.09	0.06	1.40	.161	-0.03	0.21	.11
National satisfaction	-0.05	0.04	-1.32	.186	-0.12	0.02	-.09
Perceived similarity	0.18	0.06	3.17	.002	0.08	0.30	.33
Judgment of the attack—Ingroup condition (R^2 = .10)							
National narcissism	0.19	0.08	2.48	.013	0.05	0.36	.26
National satisfaction	-0.15	0.05	-3.35	.001	-0.25	-0.07	-.30
Perceived similarity	0.12	0.06	2.23	.026	0.03	0.25	.24
Perceived similarity (R^2 = .26)							
National narcissism	0.35	0.13	2.77	.006	0.11	0.60	.24
National satisfaction	0.31	0.08	3.91	<.001	0.15	0.46	.31

Table S44

Indirect and Total Effects for the Mediation on Judgement of the Attack in the American Sample

Effect	*B*	*SE(B)*	*z*	*p*	95%CI		β
Indirect effects							
National narcissism → Similarity → Judgment of the attack—Ukraine condition (Indirect NN U)	0.06	0.03	2.39	.017	0.02	0.12	.08
National narcissism → Similarity → Judgment of the attack—Ingroup condition (Indirect NN I)	0.04	0.02	1.97	.048	0.01	0.09	.06
Contrast 1: Indirect NN U - Indirect NN I	0.02	0.02	1.28	.201	-0.01	0.05	.02
National satisfaction → Similarity → Judgment of the attack—Ukraine condition (Indirect NS U)	0.06	0.03	2.12	.034	0.02	0.12	.10
National satisfaction → Similarity → Judgment of the attack—Ingroup condition (Indirect NS I)	0.04	0.02	1.69	.091	0.01	0.10	.08
Contrast 2: Indirect NS U - Indirect NS I	0.02	0.01	1.27	.206	-0.01	0.05	.03
Total effects							
National narcissism → Judgment of the attack—Ukraine condition	0.15	0.06	2.38	.018	0.03	0.27	.19
National narcissism → Judgment of the attack—Ingroup condition	0.23	0.08	3.06	.002	0.10	0.40	.32
National satisfaction → Judgment of the attack—Ukraine condition	0.01	0.04	0.18	.854	-0.06	0.08	.01
National satisfaction → Judgment of the attack—Ingroup condition	-0.11	0.04	-2.72	.007	-0.20	-0.03	-.23

On Social Judgment of Putin

The same SEM conducted on the judgment of Putin had an acceptable fit, $\chi^2(214) = 876.43$, $p < .001$, CFI = .906, RMSEA = 0.088, 90%CI [0.082, 0.094], SRMR = 0.085.

Table S45

Loadings of Items on Latent Variables in the American Sample

Variable/Item	*B*	*SE(B)*	*z*	*p*	95%CI		β
Judgment of Putin—Ukraine condition							
Item 1 (competent)	1.00				1.00	1.00	.48
Item 2 (intelligent)	0.82	0.06	13.78	<.001	0.71	0.94	.40
Item 3 (friendly)	0.95	0.10	9.45	<.001	0.78	1.18	.79
Item 4 (empathetic)	0.91	0.11	8.19	<.001	0.71	1.15	.84
Item 5 (fair)	0.88	0.12	7.27	<.001	0.66	1.13	.86
Item 6 (trustworthy)	0.80	0.13	6.13	<.001	0.57	1.08	.82
Judgment of Putin—Ingroup condition							
Item 1 (competent)	1.00				1.00	1.00	.34
Item 2 (intelligent)	0.99	0.09	10.59	<.001	0.83	1.20	.32
Item 3 (friendly)	0.98	0.18	5.53	<.001	0.75	1.44	.82
Item 4 (empathetic)	0.86	0.20	4.40	<.001	0.60	1.35	.83
Item 5 (fair)	0.98	0.23	4.31	<.001	0.66	1.53	.78
Item 6 (trustworthy)	0.77	0.19	4.14	<.001	0.50	1.22	.79
National narcissism							
Item 1 (the United States deserves special treatment)	1.00				1.00	1.00	.77
Item 2 (I will never be satisfied until the United States gets the recognition it deserves)	0.93	0.04	22.08	<.001	0.84	1.01	.80
Item 3 (It makes me angry when people criticize the United States)	1.17	0.08	14.02	<.001	1.02	1.35	.80
Item 4 (If the United States had a major say in the world, the world would be a much better place)	1.11	0.07	15.67	<.001	0.99	1.27	.81
Item 5 (Not many people seem to fully understand the importance of the United States)	1.14	0.08	13.78	<.001	0.99	1.32	.78
National satisfaction							
Item 1 (I am glad to be American)	1.00				1.00	1.00	.92
Item 2 (I think that Americans have a lot to be proud of)	0.97	0.03	36.02	<.001	0.92	1.03	.90
Item 3 (It is pleasant to be American)	0.98	0.03	31.47	<.001	0.92	1.04	.88
Item 4 (Being American gives me a good feeling)	1.08	0.03	35.45	<.001	1.02	1.14	.95
Perceived similarity							

Item 1 (Similarity with Putin)	1.00				1.00	1.00	.92
Item 2 (Similarity with Russians)	0.62	0.09	7.30	<.001	0.45	0.79	.56

Table S46

Regressions Analyses

Variable	*B*	*SE(B)*	*z*	*p*	95%CI		β
Judgment of Putin—Ukraine condition (R^2 = .19)							
National narcissism	0.10	0.06	1.74	.082	-0.01	0.21	.13
National satisfaction	-0.06	0.04	-1.42	.155	-0.14	0.02	-.11
Perceived similarity	0.22	0.06	4.03	<.001	0.12	0.34	.42
Judgment of Putin—Ingroup condition (R^2 = .18)							
National narcissism	0.09	0.05	1.82	.069	0.00	0.20	.16
National satisfaction	-0.04	0.03	-1.24	.216	-0.10	0.02	-.10
Perceived similarity	0.15	0.05	2.82	.005	0.06	0.26	.37
Perceived similarity (R^2 = .28)							
National narcissism	0.34	0.13	2.69	.007	0.10	0.60	.25
National satisfaction	0.32	0.08	4.07	<.001	0.16	0.47	.33

Table S47

Indirect and Total Effects for the Mediation on Judgment of Putin in the American Sample

Effect	*B*	*SE(B)*	*z*	*p*	95%CI		β
Indirect effects							
National narcissism → Similarity → Judgment of Putin—Ukraine condition (Indirect NN U)	0.08	0.03	2.56	.011	0.02	0.14	.10
National narcissism → Similarity → Judgment of Putin—Ingroup condition (Indirect NN I)	0.05	0.02	2.24	.025	0.01	0.10	.09
Contrast 1: Indirect NN U - Indirect NN I	0.03	0.01	1.97	.049	0.01	0.05	.01
National satisfaction → Similarity → Judgment of Putin—Ukraine condition (Indirect NS U)	0.07	0.03	2.54	.011	0.03	0.13	.14
National satisfaction → Similarity → Judgment of Putin—Ingroup condition (Indirect NS I)	0.05	0.02	2.04	.042	0.01	0.10	.12
Contrast 2: Indirect NS U - Indirect NS I	0.02	0.01	2.28	.023	0.01	0.05	.01
Total effects							
National narcissism → Judgment of Putin—Ukraine condition	0.17	0.06	2.86	.004	0.06	0.30	.24
National narcissism → Judgment of Putin—Ingroup condition	0.14	0.05	2.67	.008	0.05	0.25	.26
National satisfaction → Judgment of Putin—Ukraine condition	0.01	0.04	0.37	.709	-0.06	0.08	.03
National satisfaction → Judgment of Putin—Ingroup condition	0.01	0.03	0.32	.750	-0.04	0.07	.02

On Judgment of Russians

Judgment of Russians.

The model had an adequate fit, $X^2(57) = 203.46$, $p < .001$, CFI = .959, RMSEA = 0.081, 90%CI [0.069, 0.093], SRMR = 0.042.

Table S48

Loadings of Items on Latent Variables in the American Sample

Variable/Item	*B*	*SE(B)*	*z*	*p*	95%CI		β
National narcissism							
Item 1 (the United States deserves special treatment)	1.00				1.00	1.00	.77
Item 2 (I will never be satisfied until the United States gets the recognition it deserves)	0.93	0.04	21.82	<.001	0.84	1.01	.79
Item 3 (It makes me angry when people criticize the United States)	1.18	0.09	13.82	<.001	1.03	1.36	.80
Item 4 (If the United States had a major say in the world, the world would be a much better place)	1.12	0.07	15.52	<.001	1.00	1.28	.81
Item 5 (Not many people seem to fully understand the importance of the United States)	1.14	0.08	13.85	<.001	0.99	1.31	.78
National satisfaction							
Item 1 (I am glad to be American)	1.00				1.00	1.00	.92
Item 2 (I think that Americans have a lot to be proud of)	0.97	0.03	35.65	<.001	0.92	1.02	.90
Item 3 (It is pleasant to be American)	0.98	0.03	30.90	<.001	0.92	1.04	.88
Item 4 (Being American gives me a good feeling)	1.07	0.03	35.99	<.001	1.02	1.13	.95
Perceived similarity							
Item 1 (Similarity with Putin)	1.00				1.00	1.00	.90
Item 2 (Similarity with Russians)	0.65	0.11	5.86	<.001	0.44	0.88	.57

Table S49

Regressions Analyses

Variable	*B*	*SE(B)*	*z*	*p*	95%CI		β
Judgment of Russians—Ukraine condition (R^2 = .04)							
National narcissism	-3.70	1.75	-2.11	.035	-7.20	-0.38	-.18
National satisfaction	1.65	1.20	1.37	.169	-0.79	3.96	.11
Perceived similarity	2.61	1.29	2.03	.043	0.45	5.51	.17
Judgment of Russians —Ingroup condition (R^2 = .03)							
National narcissism	-4.76	1.94	-2.46	.014	-8.66	-1.15	-.21
National satisfaction	1.38	1.34	1.03	.305	-1.27	4.04	.09
Perceived similarity	2.30	1.33	1.73	.084	-0.11	5.12	.14
Perceived similarity (R^2 = .28)							
National narcissism	0.33	0.13	2.50	.013	0.07	0.59	.24
National satisfaction	0.32	0.08	4.10	<.001	0.16	0.47	.33

Table S50

Indirect and Total Effects of the Mediation on Judgment of Russians in the American Sample

Effect	*B*	*SE(B)*	*z*	*p*	95%CI		β
Indirect effects							
National narcissism → Similarity → Judgment of Russians—Ukraine condition (Indirect NN U)	0.87	0.49	1.78	.075	0.08	1.97	.04
National narcissism → Similarity → Judgment of Russians—Ingroup condition (Indirect NN I)	0.76	0.50	1.52	.129	-0.06	1.89	.03
Contrast 1: Indirect NN U - Indirect NN I	0.10	0.26	0.40	.690	-0.39	0.68	.01
National satisfaction → Similarity → Judgment of Russians—Ukraine condition (Indirect NS U)	0.83	0.51	1.64	.101	0.11	2.07	.06
National satisfaction → Similarity → Judgment of Russians—Ingroup condition (Indirect NS I)	0.73	0.51	1.45	.148	-0.03	1.91	.05
Contrast 2: Indirect NS U - Indirect NS I	0.10	0.26	0.38	.705	-0.37	0.71	.01
Total effects							
National narcissism → Judgment of Russians—Ukraine condition	-2.83	1.68	-1.68	.093	-6.29	0.27	-.14
National narcissism → Judgment of Russians—Ingroup condition	-4.00	1.88	-2.13	.033	-7.86	-0.46	-.17
National satisfaction → Judgment of Russians—Ukraine condition	2.49	1.11	2.24	.025	0.36	4.65	.17
National satisfaction → Judgment of Russians—Ingroup condition	2.11	1.22	1.73	.084	-0.23	4.60	.13
